# Risk of stroke in relation to degree of asymptomatic carotid stenosis: a population-based cohort study, systematic review, and meta-analysis

**DOI:** 10.1016/S1474-4422(20)30484-1

**Published:** 2021-03

**Authors:** Dominic P J Howard, Liam Gaziano, Peter M Rothwell, Oxford Vascular Study

**Affiliations:** a Wolfson Centre for Prevention of Stroke and Dementia, Nuffield Department of Clinical Neurosciences, University of Oxford, Oxford, UK; b Department of Vascular Surgery, Oxford University Hospitals NHS Trust, Oxford, UK

## Abstract

**Background:**

There is uncertainty around which patients with asymptomatic carotid stenosis should be offered surgical intervention. Although stroke rates were unrelated to the degree of stenosis in the medical-treatment-only groups in previous randomised trials, this could simply reflect recruitment bias and there has been no systematic analysis of a stenosis-risk association in cohort studies. We aimed to establish whether there is any association between the degree of asymptomatic stenosis and ipsilateral stroke risk in patients on contemporary medical treatment.

**Methods:**

We did a prospective population-based study (Oxford Vascular Study; OxVasc), and a systematic review and meta-analysis. All patients in OxVasc with a recent suspected transient ischaemic attack or stroke, between April 1, 2002, and April 1, 2017, who had asymptomatic carotid stenosis were included in these analyses. We commenced contemporary medical treatment and determined ipsilateral stroke risk in this cohort by face-to-face follow-up (to Oct 1, 2020). We also did a systematic review and meta-analysis of all published studies (from Jan 1, 1980, to Oct 1, 2020) reporting ipsilateral stroke risk in patients with asymptomatic carotid stenosis. We searched MEDLINE, Embase, and the Cochrane Central Register of Controlled Trials, and included both observational cohort studies and medical treatment groups of randomised controlled trials if the number of patients exceeded 30, ipsilateral stroke rates (or the raw data to calculate these) were provided, and were published in English.

**Findings:**

Between April 1, 2002, and April 1, 2017, 2354 patients were consecutively enrolled in OxVasc and 2178 patients underwent carotid imaging, of whom 207 had 50–99% asymptomatic stenosis of at least one carotid bifurcation (mean age at imaging: 77·5 years [SD 10·3]; 88 [43%] women). The 5-year ipsilateral stroke risk increased with the degree of stenosis; patients with 70–99% stenosis had a significantly greater 5-year ipsilateral stroke risk than did those with 50–69% stenosis (six [14·6%; 95% CI 3·5–25·7] of 53 patients *vs* none of 154; p<0·0001); and patients with 80–99% stenosis had a significantly greater 5-year ipsilateral stroke risk than did those with 50–79% stenosis (five [18·3%; 7·7–29·9] of 34 patients *vs* one [1·0%; 0·0–2·9] of 173; p<0·0001). Of the 56 studies identified in the systematic review (comprising 13 717 patients), 23 provided data on ipsilateral stroke risk fully stratified by degree of asymptomatic stenosis (in 8419 patients). Stroke risk was linearly associated with degree of ipsilateral stenosis (p<0·0001); there was a higher risk in patients with 70–99% stenosis than in those with 50–69% stenosis (386 of 3778 patients *vs* 181 of 3806 patients; odds ratio [OR] 2·1 [95% CI 1·7–2·5], p<0·0001; 15 cohort studies, three trials) and a higher risk in patients with 80–99% stenosis than in those with 50–79% stenosis (77 of 727 patients *vs* 167 of 3272 patients; OR 2·5 [1·8–3·5], p<0·0001; 11 cohort studies). Heterogeneity in stroke risk between studies for patients with severe versus moderate stenosis (p_het_<0·0001) was accounted for by highly discrepant results (p_diff_<0·0001) in the randomised controlled trials of endarterectomy compared with cohort studies (trials: pooled OR 0·8 [95% CI 0·6–1·2], p_het_=0·89; cohorts: 2·9 [2·3–3·7], p_het_=0·54).

**Interpretation:**

Contrary to the assumptions of current guidelines and the findings of subgroup analyses of previous randomised controlled trials, the stroke risk reported in cohort studies was highly dependent on the degree of asymptomatic carotid stenosis, suggesting that the benefit of endarterectomy might be underestimated in patients with severe stenosis. Conversely, the 5-year stroke risk was low for patients with moderate stenosis on contemporary medical treatment, calling into question any benefit from revascularisation.

**Funding:**

NIHR Oxford Biomedical Research Centre, Wellcome Trust, Wolfson Foundation, and the British Heart Foundation.

## Introduction

Two large randomised controlled trials have demonstrated the benefits of carotid endarterectomy for prevention of stroke in patients with a recent symptomatic carotid stenosis,^1^, ^2^ with the observed benefit being highly dependent on the degree of stenosis.^3^, ^4^, ^5^, ^6^ Similar randomised controlled trials in patients with 50% or greater asymptomatic carotid stenosis have shown a more modest reduction in stroke risk after endarterectomy, compared with medical therapy alone.^7^, ^8^, ^9^, ^10^ However, recent cohort studies have reported lower risks of stroke with contemporary medical treatment alone,^11^, ^12^, ^13^ leading some to question the benefit of routine surgical intervention.^14^ Selection of patients for surgical intervention has been made difficult by the unexpected observation from the three pivotal clinical trials of endarterectomy for asymptomatic carotid stenosis that stroke rates on medical therapy, and hence the benefit of intervention, were unrelated to severity of stenosis.^7^, ^8^, ^9^, ^10^ As well as being inconsistent with findings in patients with symptomatic carotid stenosis,^1^, ^2^ this observation is at odds with previous cohort studies of patients with asymptomatic carotid stenosis,^15^, ^16^, ^17^, ^18^ and could simply reflect recruitment bias in randomised controlled trials.

**Research in context**

**Evidence before this study**

Randomised controlled trials in patients with asymptomatic carotid stenosis have shown modest reductions in stroke risk after endarterectomy compared with medical therapy alone, but there is uncertainty as to which patients should undergo surgical intervention and substantial variation in clinical practice worldwide. International guidelines state that surgical intervention might be indicated in patients with 60–99% asymptomatic stenosis who are at risk of stroke on medical therapy. However, selecting patients for surgical intervention has been made difficult by the unexpected observation, from the three pivotal randomised trials of endarterectomy for patients with asymptomatic carotid stenosis, that stroke rates on medical therapy, and hence the benefit of endarterectomy, were unrelated to severity of stenosis. This finding could simply reflect recruitment bias in clinical trials. Additionally, to our knowledge, there has been no systematic analysis of cohort studies. We therefore searched MEDLINE, Embase, and the Cochrane Central Register of Controlled Trials (from inception to Oct 1, 2020) to identify studies of prognosis in patients with non-operated asymptomatic carotid stenosis (defined as 50–99% atherosclerotic narrowing of the carotid bifurcation lumen or extracranial part of the internal carotid artery without ipsilateral carotid territory symptoms in the previous 6 months) on medical treatment alone. We evaluated observational cohort studies, medical treatment groups of randomised trials, and international guidelines.

**Added value of this study**

In this population-based study of patients with asymptomatic carotid stenosis who were on contemporary medical therapy, and in the accompanying systematic review and meta-analysis of cohort studies and medical treatment groups of randomised controlled trials, we show that ipsilateral stroke risk was highly dependent on the degree of stenosis, with the stroke risk being less than 5% after 5 years on contemporary medical therapy for patients with moderate stenosis, but approximately 15% in patients with severe stenosis. The strong association between the degree of asymptomatic carotid artery stenosis and ipsilateral stroke risk fits well with the proposed synergistic effect of flow reduction and embolisation in the pathogenesis of stroke distal to the carotid plaque.

**Implications of all the available evidence**

Although randomised controlled trials are the gold standard for quantifying the effects of an intervention, they are nonetheless prone to selection biases when determining risk associations within treatment groups. Guidelines should consider this issue when setting criteria for selecting patients for an intervention. Despite advances in medical therapy, the stroke risk for patients with high-grade asymptomatic carotid stenosis remains high, suggesting that the benefit of the surgical intervention might be underestimated in current guidelines. Conversely, up to 5 years of follow-up data from patients on contemporary medical therapy for moderate stenosis suggest that stroke risk is low in this population, calling into question any benefit from revascularisation.

Although randomised controlled trials are the gold standard for quantifying the effects of an intervention, they might not be reliable for determining risk associations within treatment groups.^19^ Recruitment of patients with asymptomatic carotid stenosis into randomised controlled trials required clinical equipoise between medical therapy and surgical intervention. When these clinical trials were done, there was a strong expectation that the severity of stenosis would predict stroke risk, so trial investigators might have randomly assigned patients with severe stenosis who they considered to be otherwise relatively low risk, with higher-risk patients undergoing endarterectomy outside the trial setting. Moreover, patients with moderate stenosis, who were generally considered to have a low risk of stroke, might have been more likely to be recruited if their physician felt that they had a higher than standard stroke risk on medical therapy alone. Thus, there could have been a differential recruitment bias in relation to the severity of stenosis, which would undermine any expected risk association in trial cohorts. Indeed, such risk-related recruitment was suggested in the protocol of the largest trial, which recommended recruitment of patients with 50% stenosis if they had soft plaques.^10^

On the basis of evidence from randomised controlled trials, current guidelines have omitted the degree of stenosis as a selection factor for surgical intervention in asymptomatic carotid disease,^20^, ^21^ and uncertainties about patient selection might partly explain substantial variations in clinical practice,^22^, ^23^, ^24^ with more than 75% of all endarterectomies in the USA done in patients with asymptomatic disease, compared with 7% in the UK.^23^, ^24^ We aimed to determine the association between the degree of asymptomatic stenosis and stroke risk by studying a population-based cohort of patients with asymptomatic carotid stenosis who had a transient ischaemic attack (TIA) or stroke and were on contemporary medical treatment. We also did a systematic review and meta-analysis of all studies comprising patients with medically treated asymptomatic stenosis, comparing trial cohorts and observational studies and taking into account other potentially confounding factors (eg, current smoking status, statin use, and duration of study follow-up).

## Methods

### Study design and participants

The Oxford Vascular Study (OxVasc)^25^ is a population-based study of all acute vascular events in a population of 92 748 individuals registered with more than 100 primary-care physicians in nine general practices in Oxfordshire, UK.

All patients with a recent suspected TIA or ischaemic stroke, who were referred for carotid imaging and found to have an asymptomatic carotid bifurcation stenosis from April 1, 2002, to April 1, 2017, were consecutively considered for inclusion in this analysis. Patients with a National Institute of Health Stroke Scale (NIHSS) score greater than 5 at first assessment were excluded to facilitate high rates of face-to-face follow-up and high rates of compliance with contemporary medical treatment, and to better reflect patient groups for whom carotid intervention is commonly considered.

Written informed consent or assent from family members was obtained for all participants for the study interview and follow-up, including ongoing review of primary care and hospital records, and death certificate data. OxVasc was approved by the Oxfordshire Research Ethics Committee (OREC A: 05/Q1604/70).

### Procedures

Detailed methods of OxVasc have been reported previously.^25^ Multiple overlapping methods were used for ascertainment of all individuals with suspected TIA or stroke. These included a daily, rapid access clinic to which participating general practitioners and the local emergency department refer individuals with suspected TIA or minor stroke; daily searches of admissions to the medical, stroke, neurology, vascular, and other relevant wards of the major regional centre; daily searches of the local emergency department attendance register; daily searches of in-hospital death records via the bereavement office; monthly searches of all death certificates and coroner's reports for out-of-hospital deaths; monthly searches of general practitioner diagnostic coding and hospital discharge codes; and monthly searches of all brain and vascular imaging referrals.

A detailed clinical history and examination was taken for all patients referred or ascertained to have possible cerebrovascular events. Demographic data and risk factors were collected from face-to-face interviews by study physicians as soon as possible after referral. Stroke and TIA were defined according to WHO criteria (acute onset of neurological deficit, persisting for >24 h in case of a stroke, or for <24 h in case of a TIA), with all cases reviewed as soon as possible after presentation by the same senior neurologist (PMR) throughout the study. All data on medical history and medication were cross-referenced with primary-care records.

All patients were prescribed contemporary medical treatment, including antiplatelet and statin therapy, most commonly atorvastatin 40–80 mg daily, unless contraindicated. Antihypertensive medication was initiated or increased in all patients with blood pressure higher than 130/80 mm Hg at baseline or during follow-up. All patients were given lifestyle advice, particularly on smoking cessation if relevant.

Asymptomatic carotid stenosis was defined as a 50% or greater reduction in the diameter of the carotid artery without a previous stroke or TIA in the territory of the asymptomatic carotid artery. Carotid occlusions, isolated proximal common carotid stenoses, and intracranial internal carotid stenoses were excluded. In patients who had a posterior circulation TIA or stroke, with asymptomatic stenosis of both carotid arteries, the artery with the most severe stenosis was included.

Patients were followed up at 1, 6, 12, and 24 months and at 5 and 10 years by a study nurse or physician, up to Oct 1, 2020. All cerebrovascular events that occurred during follow-up were identified by ongoing daily case ascertainment within OxVasc. All patients with neurological events were reassessed and reviewed by the senior neurologist.

From 2002 to 2010, carotid stenosis was detected in patients through carotid duplex ultrasound (ATL Ultramark HDI 5000 scanner; Bothell, WA, USA). From 2010 to 2017, contrast-enhanced magnetic resonance angiographic imaging (Siemens Verio 3 Tesla scanner with head and neck coil; Oxford, UK) was used as a first-line screen, with stenosis subsequently confirmed and quantified with carotid artery ultrasound (Philips Epiq 7 scanner; Eindhoven, Noord-Brabant, Netherlands). Carotid duplex ultrasound scans were done at an accredited vascular laboratory (Regional Vascular Unit of John Radcliffe Hospital, Oxford, UK) according to current guidelines.^26^, ^27^ The ultrasound protocol included B-mode imaging (transverse and longitudinal plane) of the proximal, mid, and distal common carotid artery; carotid bifurcation; and proximal, mid, and distal extracranial internal carotid artery. The presence of carotid plaques and plaque morphology were documented. Peak systolic velocity and end-diastolic velocity were measured by pulsed-wave Doppler analysis on the common carotid artery and extracranial internal carotid artery bilaterally. Duplex data were used for designation of stenosis in our analyses, with calibration to the North American Symptomatic Carotid Endarterectomy Trial (NASCET) method of measuring the degree of stenosis.^28^

### Search strategy and selection criteria

We did a systematic review and meta-analysis of studies reporting the risk of ipsilateral stroke in patients with asymptomatic carotid stenosis receiving medical therapy, with particular reference to the degree of carotid stenosis. We followed PRISMA and MOOSE reporting guidelines (appendix pp 1–3).^29^

We did a literature search of MEDLINE, Embase, and the Cochrane Central Register of Controlled Trials (from inception to Oct 1, 2020) to identify studies of prognosis in patients with non-operated asymptomatic carotid stenosis (defined as 50–99% atherosclerotic narrowing of the carotid bifurcation lumen or extracranial part of the internal carotid artery without ipsilateral carotid territory symptoms in the previous 6 months) while on medical treatment alone. We included both observational cohort studies and the medical treatment groups of randomised trials. Studies published from Jan 1, 1980, to Oct 1, 2020, were eligible for inclusion, provided the number of patients studied exceeded 30, ipsilateral stroke rates (or the raw data to calculate these) were provided, and the studies were published in English.

In electronic database searches, we combined search terms for carotid stenosis, relevant intervention or medical treatment, and outcome using the combination of terms shown in the appendix (p 4). Additionally, we reviewed the bibliographies of all papers identified and relevant systematic and narrative reviews. In cases of multiple publications from the same cohort, the first study to be published and reporting the required data was used, unless the number of patients increased in a later publication, in which case the study reporting the larger cohort was used.

Two reviewers (DPJH and LG) independently searched, screened, and selected all studies. The full text of abstracts considered potentially relevant by any reviewer were retrieved. Studies reporting mixed cohorts of patients (with symptomatic and asymptomatic carotid artery stenosis) were included only if results were stratified according to symptom status. Discrepancies were resolved by consensus with a third investigator (PMR).

DPJH and LG extracted data from each study into electronic tables, including data on study design, funding, setting, population characteristics, the definition of carotid stenosis, the imaging modality used, and related diagnostic and quality assurance criteria for determining the degree of stenosis, and details about medical therapies. For each outcome of interest, we recorded the outcome definitions used, the baseline screening methods, the frequency and duration of follow-up, and the methods for outcome ascertainment. For all studies, the degree of stenosis was corrected to meet the NASCET criteria and measurement of carotid stenosis.^28^

We assessed the risk of bias and the quality of reporting of individual studies with STROBE criteria. The STROBE statement for OxVasc is summarised in the appendix (pp 5–6).

### Statistical analysis

For the OxVasc study, baseline clinical characteristics and initial medical treatment were compared between patients with moderate stenosis and those with severe asymptomatic stenosis. Numerical data were expressed as means (SDs) or proportions, as appropriate. Group differences in continuous parametric variables (such as age) were examined with Student's *t* test or one-way ANOVA, as appropriate. Group differences in categorical variables were examined with Fisher's exact test or χ^2^ test, as appropriate. p values less than 0·05 were considered significant.

The risk of first ipsilateral ischaemic event (TIA or stroke) during follow-up distal to an asymptomatic carotid stenosis was ascertained from the date of vascular imaging. Patients undergoing carotid intervention were censored at the time of the procedure. Kaplan-Meier analysis survival curves were plotted for each outcome during the first 5 years of follow-up in patients with moderate versus severe asymptomatic stenosis, and the significance of the differences in risk was determined by the log-rank test. The difference in 5-year risk was estimated with Cox proportional hazards modelling in which the relative hazards were not time dependent. Analyses were done with SPSS, version 25.0.

For the systematic review and meta-analysis, we calculated the average annual ipsilateral stroke rate as the number of ipsilateral strokes per 100 person-years of observation. If the total patient-years of observation was not reported, we calculated this as the number of patients included multiplied by the mean follow-up time. Pooled risk estimates were obtained by the Mantel-Haenszel method, with 95% CIs calculated to allow for extra-binomial variation, since standard methods of calculating confidence intervals can produce artificially narrow intervals if there is heterogeneity of risk between different studies. The heterogeneity of risk estimates across studies was assessed with χ^2^ tests.

We first determined the pooled estimate of the risk of ipsilateral ischaemic stroke for all eligible studies for patients with 50–99% carotid stenosis. Subsequent meta-analyses were restricted to those studies that included patients with 50–99% stenosis and reported stroke risk stratified by the degree of carotid stenosis at baseline, such that risks were reported, or could be derived, for patients with moderate versus severe stenosis, defined in most studies with a cutoff point of either 70% or 80%. As the preferred cutoff point used to define severe asymptomatic stenosis is under debate, we used both cutoff points in the meta-analysis.

For each study we calculated the odds ratio (OR) and 95% CI for the risk of ipsilateral ischaemic stroke during follow-up for patients with moderate versus severe stenosis (separately for the 70% and 80% cutoff points), and combined estimates by fixed-effects meta-analysis (Mantel-Haenszel-Peto method) when there was limited heterogeneity between studies (χ^2^ test p<0·05). Otherwise, we did a random-effects analysis. The pooled analysis was primarily stratified by the nature of the study design—an observational cohort study versus the medical treatment group of randomised trials. In the event of heterogeneity between studies in the association of stenosis with stroke risk, further sensitivity analyses were done with stratification by previous statin therapy (<50% *vs* ≥50% of patients on statin therapy), inclusion of patients with previous cerebrovascular events (no previous cerebrovascular events in any territory *vs* previous events), year of study (publication date before 2000 *vs* 2000 or later), and grading of study quality based on STROBE criteria and key criteria required for meta-analysis, including the following items: study objectives stated clearly; patient selection criteria stated clearly; patients enrolled consecutively without predetermined selection; interventions adequately described; outcome definitions provided; rate of dropout or crossover to endarterectomy or stenting of less than 20%, and outcome ascertainment by a neurologist. We graded each study as low, medium, or high based on these criteria. Potential publication bias in relation to the association of stenosis with stroke risk was assessed with an Egger funnel plot.

We did further pooled analyses of annual ipsilateral stroke rates in patients with moderate versus severe stenosis stratified by time period (decades), statin use, current smoking prevalence, and median length of follow-up. We also derived the proportion of patients recruited with moderate versus severe stenosis as a surrogate measure of the generalisability of the stenosis pattern in the study cohort to that expected in the underlying population, based on an expected ratio from population-screening studies of 2·5:1·0 for 50–69% stenosis versus 70–99% stenosis.^30^ We assessed the heterogeneity of the stenosis pattern across studies with χ^2^ tests and used meta-regression to relate the proportion of patients with moderate to severe stenosis to the relative odds of ipsilateral ischaemic stroke in the two stenosis groups, using the Stata command metareg, Stata version IC/12·1.

### Role of the funding source

The funders of the study had no role in study design, data collection, data analysis, data interpretation, or writing of the report.

## Results

Of 2354 eligible patients consecutively enrolled in OxVasc from April 1, 2002, to April 1, 2017, 2178 (92%) had carotid imaging. Initial imaging was done with ultrasound for 1591 (73%) of 2178 patients, and with contrast-enhanced magnetic resonance angiogram for 587 (27%) of 2178. Patients with a disabling or fatal stroke (95 [54%] of 176) or dementia (81 [46%] of 176) were not imaged.

Of the 2178 imaged patients, 461 had 50% or greater stenosis of at least one carotid bifurcation, of whom 243 had 50–99% symptomatic carotid stenosis and 11 had an occlusion, and 207 had 50–99% asymptomatic carotid stenosis and eight had an occlusion.

The 207 patients with 50–99% asymptomatic carotid stenosis had a mean age at imaging of 77·5 (SD 10·3) years and 200 (97%) had cardiovascular risk factors. 92 (44%) were on statin therapy before recruitment and 115 (56%) were on antiplatelet or anticoagulant treatment (table). After recruitment, contemporary medical therapy was commenced and maintained in 189 survivors at 12 months’ follow-up, with 179 (95%) patients on anti-thrombotic treatment, 169 (89%) on a statin, and 168 (89%) on at least one blood-pressure-lowering drug.

**Table:** Baseline clinical characteristics and medical therapy after diagnosis and at 12 months' follow-up in patients with asymptomatic carotid stenosis in the Oxford Vascular Study, stratified by degree of stenosis

		**Total (n=207)**	**50–69% stenosis (n=154)**	**70–99% stenosis (n=53)**	**p value**
Mean age, years		77·5 (10·3)	77·9 (9·7)	76·2 (11·7)	0·28
Sex					
	Female	88 (43%)	71 (46%)	17 (32%)	0·08
	Male	119 (57%)	83 (54%)	36 (68%)	..
Previous vascular disease					
	TIA or stroke	182 (88%)	138 (90%)	44 (83%)	0·51
	Coronary artery disease	66 (32%)	49 (32%)	17 (32%)	0·97
	Cardiac failure	24 (12%)	15 (10%)	9 (17%)	0·16
	Peripheral arterial disease	47 (23%)	30 (19%)	17 (32%)	0·06
Cardiovascular risk factors					
	Any	200 (97%)	149 (97%)	51 (96%)	0·86
	Current smoker	34 (16%)	21 (14%)	13 (25%)	0·07
	Ever smoked	141 (68%)	101 (66%)	40 (75%)	0·18
	Hypertension	164 (79%)	123 (80%)	41 (77%)	0·70
	Diabetes	42 (20%)	29 (19%)	13 (25%)	0·37
	Hyperlipidaemia	109 (53%)	83 (54%)	26 (49%)	0·54
	Atrial fibrillation	51 (25%)	40 (26%)	11 (21%)	0·45
Premorbid medication					
	Statin	92 (44%)	73 (47%)	19 (36%)	0·14
	Antiplatelet or anticoagulant	115 (56%)	85 (55%)	30 (57%)	0·50
	Antihypertensives	156 (75%)	117 (76%)	39 (74%)	0·73
Post-diagnosis medication					
	Statin	178 (86%)	136 (88%)	42 (79%)	0·19
	Antiplatelet or anticoagulant	199 (96%)	147 (95%)	52 (98%)	0·86
	Antihypertensives	181 (87%)	135 (88%)	46 (87%)	0·91
12-month follow-up*					
	Statin	169/189 (89%)	138/150 (92%)	31/39 (79%)	0·15
	Antiplatelet or anticoagulant	179/189 (95%)	142/150 (95%)	37/39 (95%)	0·97
	Antihypertensives	168/189 (89%)	133/150 (89%)	35/39 (90%)	0·91
Ipsilateral events†					
	TIA or stroke	16 (1·34)	6 (0·89)	10 (13·7)	<0·0001
	Stroke	8 (0·66)	2 (0·22)	6 (2·08)	<0·0001

Data are n (%) or mean (SD). TIA=transient ischaemic stroke.

* In 189 survivors.

† Number of events during follow-up (rate per 100 person-years).

Median follow-up was 5·9 years (IQR 3·4–8·7; 1316 patient-years), and all patients were followed up to Oct 1, 2020. Ten (5%) patients had an endarterectomy for asymptomatic stenosis during follow-up (median time to surgery 2·6 years [IQR 0·4–4·9]), and seven had an endarterectomy after an ipsilateral TIA or stroke (median time to surgery 7·0 years [4·4–8·7]). No patients had carotid angioplasty or stenting.

**Figure 1:**
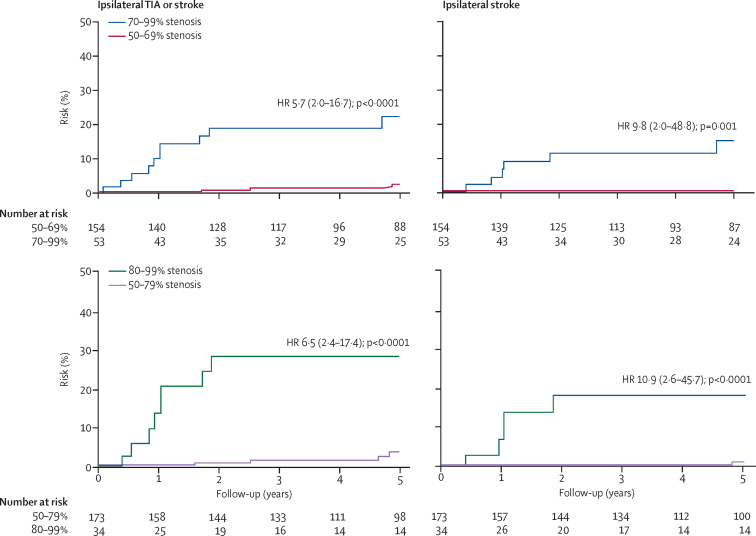
Risk of recurrent vascular events by degree of asymptomatic ipsilateral carotid stenosis in 207 patients with asymptomatic carotid stenosis in the Oxford Vascular Study The exact range and cutoff points used to report the stenosis and risk data in trials and previous studies vary, with some defining severe stenosis as 70–99% and some as 80–99%. We therefore analysed both cutoff points in the Oxford Vascular Study. TIA=transient ischaemic attack. HR=hazard ratio.

There were 16 ischaemic events (eight strokes and eight TIAs) in the territory of a 50–99% asymptomatic stenosis before any endarterectomy during follow-up (table). The average annual risk of any ipsilateral carotid territory ischaemic stroke was 0·66 per 100 person-years (95% CI 0·29–1·30) and that of any ipsilateral carotid territory event (TIA or stroke) was 1·34 per 100 person-years (0·77–2·18). The 5-year risk of ipsilateral ischaemic stroke was significantly greater in patients with 70–99% stenosis than in patients with 50–69% stenosis (six [14·6%; 95% CI 3·5–25·7] of 53 patients *vs* none of 154; p<0·0001), significantly greater in patients with 80–99% stenosis than in those with 60–79% stenosis (five [18·3%; 7·7–29·9] of 34 patients *vs* two [2·3%; 0·0–6·8] of 76; p<0·0001), and significantly greater in patients with 80–99% stenosis than in those with 50–79% stenosis (five [18·3%; 7·7–29·9] of 34 patients *vs* one [1·0%; 0·0–2·9] of 173; p<0·0001; figure 1; appendix p 7). Results were similar when patients with atrial fibrillation at baseline were excluded (appendix p 8).

In the systematic review (the flowchart depicting the selection of studies is summarised in the appendix [p 9]), 56 studies, comprising 13 717 patients, met the inclusion criteria: 36 prospective observational cohort studies, five randomised controlled trials, and 15 retrospective cohort studies (appendix pp 10–12). The majority of cohorts were from North America (n=27) and Europe (n=21), with five from Asia, and three from Australia. The years of recruitment, observation periods and number of ipsilateral events, and the proportion of study participants on statin therapy are summarised in the appendix (pp 13–14). Of the 56 studies, 11 had an upper limit of stenosis lower than 90% (eg, 50–70%).

**Figure 2:**
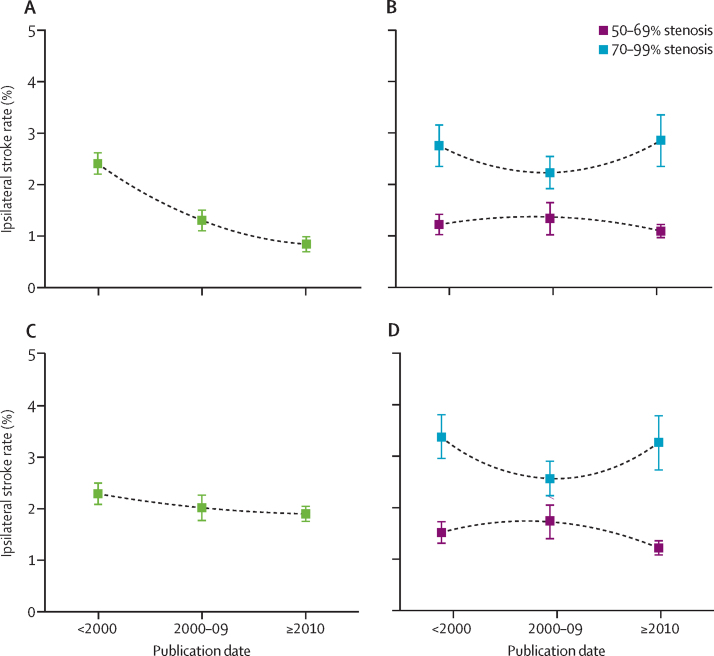
Time trends in pooled estimates of annual ipsilateral stroke rates in medically treated patients with asymptomatic carotid stenosis, stratified by degree of baseline carotid artery stenosis, in a systematic review of studies published after 1980 Data are pooled annual ipsilateral stroke rates and 95% CIs. (A) Stroke rate distal to 50–99% stenosis in all studies (n=56). (B) Stroke rate distal to 50–69% versus 70–99% stenosis in all non-selective cohort studies reporting risk for patients with 50–69% or 70–99% stenosis (n=30). (C) Stroke rate distal to 50–99% stenosis in all non-selective cohort studies reporting risk in both patients with 50–69% stenosis and those with 70–99% stenosis (n=15). (D) Stroke rate distal to 50–69% versus 70–99% stenosis in all non-selective cohort studies reporting risk in both patients with 50–69% stenosis and those with 70–99% stenosis (n=15).

Across all studies, the pooled estimate of the annual risk of ipsilateral ischaemic stroke distal to 50–99% asymptomatic carotid stenosis fell over the three decades of analysis, from 2·38% (95% CI 2·18–2·58) in studies published before 2000, to 0·88% (0·62–1·02) after 2010 (p<0·0001; figure 2A). However, when the analysis was stratified by the degree of stenosis, including 30 studies that provided ipsilateral stroke risk stratified to some extent by the degree of asymptomatic carotid stenosis at baseline, the risk of stroke was consistently two to three times higher distal to 70–99% stenosis than to 50–69% stenosis in all three decades (figure 2B). This finding was corroborated in non-selective cohort studies that reported fully stratified stroke risk in patients with both 50–69% and 70–99% stenosis (figure 2C, D).

**Figure 3:**
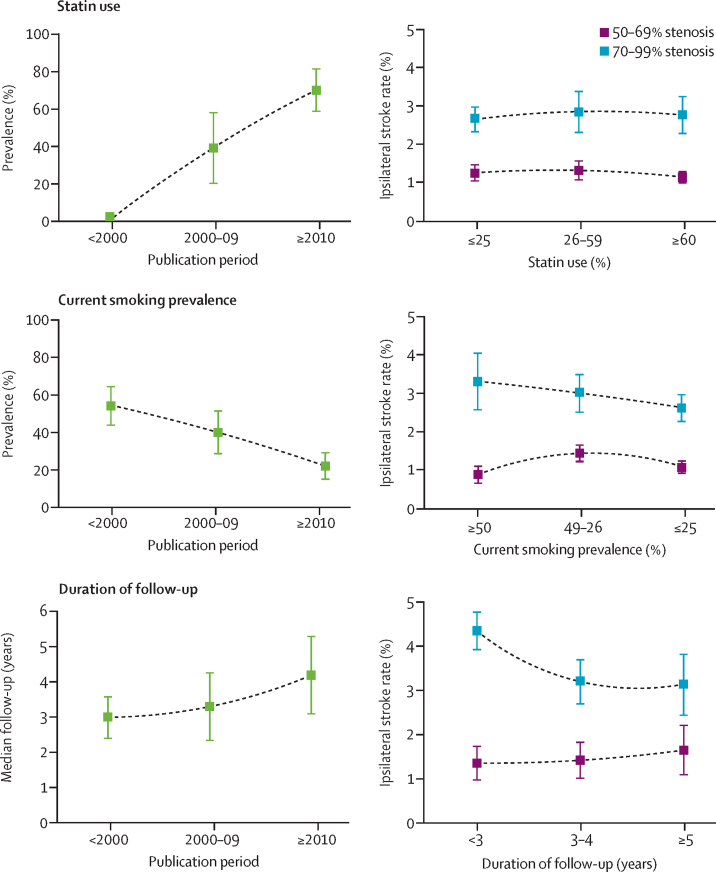
Time trends in statin use, current smoking prevalence, and duration of follow-up, and ipsilateral stroke rates stratified by confounding factor Data are pooled annual ipsilateral stroke rates and 95% CIs. Data are from all non-selective cohort studies (n=30) reporting stroke risk for patients with 50–69% or 70–99% stenosis. Time trends for medically treated patients with asymptomatic carotid stenosis are shown on the left.

In the 30 stenosis-stratified cohort studies, statin use increased over time (p<0·0001), whereas the prevalence of current smoking decreased (p<0·0001), and the median duration of study follow-up increased (p=0·0063; figure 3). However, the excess ipsilateral stroke risk distal to severe versus moderate carotid stenosis was independent of these factors (figure 3).

**Figure 4:**
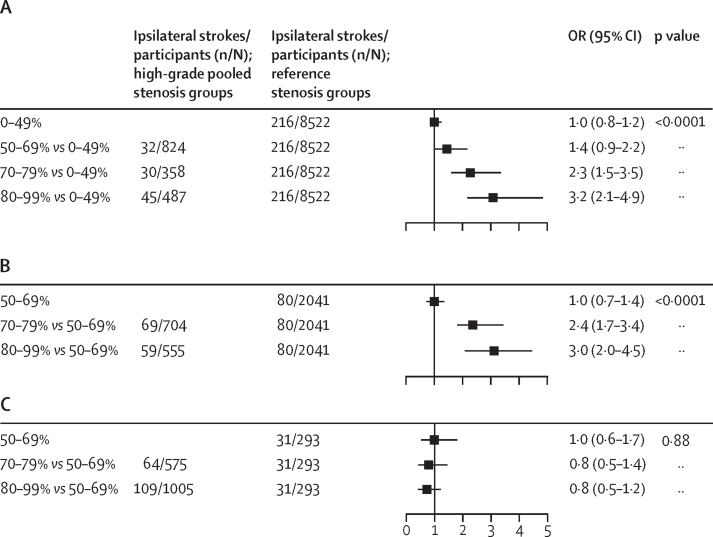
Odds of ipsilateral stroke by degree of stenosis in medically treated patients with asymptomatic carotid stenosis (A) Five cohort studies reporting stroke risk for patients with 0–99% stenosis. (B) Seven cohort studies reporting stroke risk for patients with 50–99% stenosis. (C) The medical treatment group of randomised trials. OR=odds ratio.

23 studies, including the medical treatment groups of the three randomised controlled trials, reported fully stratified ipsilateral stroke risk in both moderate and severe stenosis (in 8419 patients). Meta-analysis of these studies revealed a linear association of stroke risk with degree of stenosis (p<0·0001; figure 4), with a higher risk distal to 70–99% versus 50–69% stenosis (386 of 3778 patients *vs* 181 of 3806 patients; OR 2·1 [95% CI 1·7–2·5]; p<0·0001; 15 cohort studies, three trials) and 80–99% versus 50–79% stenosis (77 of 727 patients *vs* 167 of 3272 patients; 2·5 [1·8–3·5], p<0·0001; 11 cohort studies; appendix p 15), but there was significant heterogeneity between studies (p_het_<0·0001). However, this heterogeneity was accounted for by the three trials of endarterectomy for asymptomatic stenosis, which yielded highly discrepant results (p_diff_<0·0001) when compared with cohort studies (trials: pooled OR 0·8 [95% CI 0·6–1·2]; p_het_=0·89; cohorts: 2·9 [2·3–3·7]; p_het_=0·54; appendix p 15). Among cohort studies, inclusion of patients with previous cerebrovascular events in other territories did not alter the association between ipsilateral stroke risk and degree of stenosis (appendix p 15), and further sensitivity analyses in relation to previous statin therapy, year of study publication, and study quality did not explain any further heterogeneity (appendix pp 16–18).

The annual ipsilateral stroke risk in patients with 50–69% stenosis was significantly higher in trials than in cohort studies (2·33% [95% CI 1·85–2·82] in trials *vs* 1·23% [1·1–1·37] in cohorts; p<0·0001), and in patients with 70–99% stenosis the annual ipsilateral stroke risk was significantly lower in trials than in cohort studies (1·93% [1·73–2·12] in trials *vs* 2·81% [2·54–3·08] in cohorts; p<0·0001; appendix p 19). The trials also had a much higher ratio of patients with 70–99% than 50–69% stenosis (pooled ratio: 5·39 [95% CI 2·64–8·14] in trials *vs* 0·58 [0·29–0·87] in cohorts; p_diff_<0·0001). Thus, when the ratio of observed stroke risk at 70–99% versus 50–69% stenosis was plotted against the proportion of study participants with 70–99% stenosis (appendix p 20), the cohort studies and trials formed two distinct clusters (k-means cluster analysis, p_diff_<0·0001). In cohort studies, there was an 8·17% (95% CI 7·04–9·30) reduction in the proportion of patients included with severe stenosis over 20 years (p=0·0041; appendix p 20), but this reduction did not confound the excess risk of ipsilateral stroke distal to severe versus moderate stenosis (appendix p 20). An Egger funnel plot did not reveal any evidence of significant publication bias (appendix p 21).

## Discussion

In this prospective cohort study and systematic review and meta-analysis examining the ischaemic stroke risk in relation to asymptomatic carotid stenosis, we show that, although reported rates of ipsilateral stroke have fallen over time, the stroke risks in cohort studies are still highly dependent on the degree of stenosis. The medical treatment groups of the three randomised controlled trials of endarterectomy for asymptomatic carotid stenosis yielded discrepant results compared with cohort studies, probably due to selective recruitment of participants, as indicated by their substantially higher ratio of patients with severe versus moderate stenosis. We also found time trends in current smoking prevalence, statin use, median duration of study follow-up, and prevalence of severe stenosis, but the excess risk of stroke distal to severe versus moderate stenosis was independent of these factors.

That the degree of asymptomatic carotid stenosis is highly predictive of ipsilateral stroke risk is consistent with findings from patients with symptomatic carotid stenosis.^3^, ^4^, ^5^, ^6^ Yet, based on data from randomised trials and from previous reviews of studies investigating the prognosis of patients with asymptomatic carotid disease that did not stratify analyses into trial versus non-trial cohorts,^11^, ^12^, ^13^ guidelines continue to omit degree of stenosis as a selection factor for intervention in patients with asymptomatic carotid disease, and instead advocate the use of partly validated surrogate markers for patient selection, such as computerised plaque analysis, plaque echolucency, and intra-plaque haemorrhage on MRI.^20^ However, these surrogate markers are not routinely assessed in clinical practice. Selection of patients on the basis of the degree of stenosis would be more straightforward and might help to standardise international practice.

We assessed the proportion of patients enrolled with moderate versus severe stenosis as a surrogate measure of the generalisability of the study cohort on the basis of the expected ratio of 2·5:1·0 from population-screening studies.^30^ In OxVasc, and many other cohort studies, the ratio was consistent with that expected, whereas the proportion of patients with moderate stenosis was much lower in trial cohorts, indicating the potential for selection bias. We cannot ascertain the exact nature of any such bias, but given the previous expectation at the time of the trials that the severity of stenosis would predict stroke risk, trial investigators might have randomly assigned patients with severe stenosis who they considered to be otherwise relatively low risk, and enrolled patients with moderate stenosis who were thought to be otherwise high risk. Our analysis of the absolute risks of stroke in the cohort studies versus trials is consistent with this hypothesis, as is the protocol of the largest trial, which suggested recruitment of patients with 50% stenosis if they had soft plaque.^9^

Strengths of our study include the consistency of the findings in OxVasc and other cohort studies, together with the robustness of our analysis of the association of stenosis with stroke risk, stratified by potential confounders. However, some limitations should be noted. First, the number of strokes in the OxVasc analysis were small. However, as a prospective population-based cohort study with high participation rates, near-complete follow-up, and a policy of limited surgical intervention for asymptomatic carotid stenosis, this analysis was well placed to give unbiased estimates of any stenosis-stroke risk association while on contemporary medical treatment. Second, although contemporary best medical therapy was given to all patients with high rates of concordance, highly intensive medical therapy (complete smoking cessation, a Mediterranean diet, and absolute control of all risk factors) was not feasible due to the study design. Third, inclusion of patients with previous cerebrovascular events in other territories in both OxVasc and several other cohort studies might be considered a limitation. However, in the absence of widespread carotid screening programmes, this is how patients with asymptomatic carotid stenosis are most commonly identified in routine clinical practice; patients are largely diagnosed by carotid artery imaging following neurological symptoms. Moreover, we showed that the association between stenosis and stroke risk was similar in cohort studies that included only asymptomatic patients (with no previous cerebrovascular events in other territories) and those that included patients with previous events in other territories. Fourth, the majority of patients who had a stroke or TIA in OxVasc had undergone carotid imaging, but some patients with disabling or fatal strokes or dementia did not. Fifth, there were differences in the exact range and cutoff points used to report the stenosis risk data in studies included in the systematic review, with some defining severe stenosis as 70–99% and some as 80–99%. However, pooled analyses were broadly similar with either cutoff point. Finally, measurement of carotid artery stenosis is prone to error and to inter-observer variability across all imaging modalities.^31^, ^32^ In both OxVasc and in most of the cohorts included in the systematic review, stenosis was quantified with ultrasound, which has considerable inter-observer variability.^31^ In addition to observer effects, the strength of the association between the degree of stenosis and stroke risk in symptomatic patients is also highly dependent on the quality of angiographic imaging,^33^ and similar problems might exist for ultrasound assessment. However, any shortcomings in either imaging or observer accuracy would be expected to reduce the strength of the association between degree of stenosis and stroke risk, and make our findings more conservative.

Our findings have implications for understanding the mechanisms of stroke and for future research. As observed in patients with symptomatic disease,^6^, ^33^ the degree of asymptomatic carotid artery stenosis also appears to be strongly associated with ipsilateral stroke risk, which fits well with the proposed synergistic effect of flow reduction and embolisation in the pathogenesis of stroke in relation to carotid plaques.^34^ In view of the strong association observed between the degree of stenosis and stroke risk, research investigating other surrogate markers associated with stroke risk in asymptomatic patients must be fully adjusted for the degree of carotid stenosis. Additionally, our findings are an important reminder that, although randomised controlled trials are the gold standard for quantifying the effects of an intervention, they might not be suitable for determining risk associations within treatment groups and so should not be used for epidemiological analyses.

Our findings have implications for clinical practice. Contrary to assumptions in current guidelines, the stroke risk in cohort studies was highly dependent on the degree of asymptomatic stenosis, being less than 5% after 5 years on contemporary medical therapy for moderate stenosis, but approximately 15% for patients with severe stenosis. In Europe, intervention rates for high-grade stenosis might be too low, whereas in the USA the intervention rates for moderate stenosis might be too high, as recent data from the American College of Surgeons National Surgical Quality Improvement Program confirm that 25% of all endarterectomies for asymptomatic disease were done in patients with moderate stenosis.^35^

Our findings also highlight the importance of accuracy in measurement of stenosis in routine practice, within certified vascular units. Finally, our findings support the notion of surveillance for moderate asymptomatic carotid artery stenosis in appropriate subgroups, since the predictive value of the baseline severity of stenosis was diluted in studies with a longer follow-up, potentially due to progression of moderate stenosis over time.

In conclusion, our findings confirm previous reports that the risk of stroke distal to asymptomatic carotid stenosis has declined over the decades.^10^, ^11^, ^12^ However, we have shown that stroke risk remains high for patients with high-grade stenosis on contemporary medical therapy, suggesting that the benefits of surgical intervention might be underestimated. Conversely, stroke risk was low for patients with moderate stenosis on contemporary medical therapy, calling into question any benefit of revascularisation.

## Data sharing

Requests for access to the data reported in this Article will be considered by the corresponding author.
