# Correction to *Lancet Neurol* 2021; 20: 193–20

**DOI:** 10.1016/S1474-4422(21)00076-4

**Published:** 2021-05

*Howard DPJ, Gaziano L, Rothwell PM, on behalf of the Oxford Vascular Study. Risk of stroke in relation to degree of asymptomatic carotid stenosis: a population-based cohort study, systematic review, and meta-analysis.* Lancet Neurol *2021;* 20: *193–202*—In the table of this Article, the rate (per 100 person-years) of transient ischaemic attack or stroke should have been 0·66 in the 50–69% stenosis group and 3·51 in the 70–99% stenosis group. And the second paragraph of the Results section should read as follows: “Of the 2178 imaged patients, 469 had 50% or greater stenosis of at least one carotid bifurcation, of whom 243 had 50–99% symptomatic carotid stenosis and 11 had an occlusion, and 207 had 50–99% asymptomatic carotid stenosis and eight had an occlusion.” These corrections have been made to the online version as of March 16, 2021.

