# Supplementary material for: Risk of stroke in relation to degree of asymptomatic carotid stenosis: a population-based cohort study, systematic review, and meta-analysis
Source: Lancet Neurol. 2021 Mar;20(3):193–202. doi: 10.1016/S1474-4422(20)30484-1 (PMC7889579; doi:10.1016/S1474-4422(20)30484-1)

THE LANCET
Neurology

Supplementary appendix

This appendix formed part of the original submission and has been peer reviewed. We post it as supplied by the authors.

Supplement to: Howard DPJ, Gaziano L, Rothwell PM, on behalf of the Oxford Vascular Study, et al. Risk of stroke in relation to degree of asymptomatic carotid stenosis: a population-based cohort study, systematic review, and meta-analysis. *Lancet Neurol* 2021; **20:** 193–202.

Supplementary Material

Supplementary Appendix 1. Reporting checklist for meta-analysis of observational studies.

Based on the MOOSE guidelines.

		Reporting Item	Page Number
**Title**			
	#1	Identify the study as a meta-analysis of observational research	1
**Abstract**			
	#2	Provide a structured summary including, as applicable: background; objectives; data sources; study eligibility criteria, participants, and interventions; study appraisal and synthesis methods; results; limitations; conclusions and implications of key findings; systematic review registration number (From PRISMA checklist)	3
**Background**			
	#3a	Problem definition	2
	#3b	Hypothesis statement	5
	#3c	Description of study outcomes	2
	#3d	Type of exposure or intervention used	5
	#3e	Type of study designs used	5
	#3f	Study population	5
**Methods**			
Search strategy	#4a	Qualifications of searchers (eg, librarians and investigators)	7-8
Search strategy	#4b	Search strategy, including time period included in the synthesis and keywords	7
Search strategy	#4c	Effort to include all available studies, including contact with authors	7, Appendix Figure 3
Search strategy	#4d	Databases and registries searched	7

Search strategy	#4e	Search software used, name and version, including special features used (eg, explosion)	7, Appendix 2
Search strategy	#4f	Use of hand searching (eg, reference lists of obtained articles)	7-8, Appendix 4
Search strategy	#4g	List of citations located and those excluded, including justification	7-8, Appendix Figure 3
Search strategy	#4h	Method of addressing articles published in languages other than English	7-8
Search strategy	#4i	Method of handling abstracts and unpublished studies	7-8
Search strategy	#4j	Description of any contact with authors	7-8
	#5a	Description of relevance or appropriateness of studies gathered for assessing the hypothesis to be tested	Appendix Table 1
	#5b	Rationale for the selection and coding of data (eg, sound clinical principles or convenience)	7-9
	#5c	Documentation of how data were classified and coded (eg, multiple raters, blinding, and interrater reliability)	7-9
	#5d	Assessment of confounding (eg, comparability of cases and controls in studies where appropriate)	7-9
	#5e	Assessment of study quality, including blinding of quality assessors; stratification or regression on possible predictors of study results	7-9, Appendix Table 1
	#5f	Assessment of heterogeneity	9, 11
	#5g	Description of statistical methods (eg, complete description of fixed or random effects models, justification of whether the chosen models account for predictors of study results, dose-response models, or cumulative meta-analysis) in sufficient detail to be replicated	8-9
	#5h	Provision of appropriate tables and graphics	Appendix Table 1, Figure 2
**Results**			
	#6a	Graphic summarizing individual study estimates and overall estimate	Figures 4, Appendix Figures 3
	#6b	Table giving descriptive information for each study included	Appendix Table 1
	#6c	Results of sensitivity testing (eg, subgroup analysis)	Figure 2-3, Appendix Figures 4-8
	#6d	Indication of statistical uncertainty of findings	10-11

Discussion

	#7a	Quantitative assessment of bias (eg. publication bias)	10-12, Appendix Figure 9
	#7b	Justification for exclusion (eg, exclusion of non–English-language citations)	8-9, 10-13
	#7c	Assessment of quality of included studies	12-13, Appendix Table 1

Conclusion

	#8a	Consideration of alternative explanations for observed results	12-13
	#8b	Generalization of the conclusions (ie, appropriate for the data presented and within the domain of the literature review)	12-13
	#8c	Guidelines for future research	12-13
	#8d	Disclosure of funding source	14

Supplementary Appendix 2

PUBMED/MEDLINE(R) 1946 TO PRESENT - ACCESSED 1st OCTOBER 2020
EMBASE (OVID) 1974 TO PRESENT- ACCESSED 1st OCTOBER 2020

1. exp Carotid Stenosis/
2. Plaque, Atherosclerotic/
3. (carotid adj3 (stenos$ or athero$ or narrow$ or constrict$ or obstruct$ or ulcer$ or plaque$ or occlus$ or bruit$)).tw.
4. exp Stroke/
5. Stroke$.tw.
6. cerebrovascular.tw.
7. ((brain or vascular or lacunar or venous or cerebral or isch?emic) adj2 (accident$ or infarct$ or event$ or attack$)).tw.
8. (cva or cvas).tw.
9. or/4-8

THE COCHRANE LIBRARY WILEY - ACCESSED 1st OCTOBER 2020
#1 MeSH descriptor: [Carotid Stenosis] explode all trees
#2 MeSH descriptor: [Plaque, Atherosclerotic] explode all trees
#3 (carotid near/3 (athero* or stenos* or ulcer* or plaque* or narrow* or obstruct* or occlus* or constrict* or bruit*))
#4 #1 or #2 or #3
#5 MeSH descriptor: [Stroke] explode all trees
#6 Stroke*
#7 cerebrovascular
#8 cva or cvas
#9 #4 or #5 or #6 or #7 or #8

Supplementary Appendix 3. STROBE Statement—Checklist of items that should be included in reports of *cohort studies*

Section/Topic	Item #	Recommendation	Reported on page #
**Title and abstract**	1	(*a*) Indicate the study's design with a commonly used term in the title or the abstract	2
		(*b*) Provide in the abstract an informative and balanced summary of what was done and what was found	2
**Introduction**			
Background/rationale	2	Explain the scientific background and rationale for the investigation being reported	3-4
Objectives	3	State specific objectives, including any prespecified hypotheses	3-4
**Methods**			
Study design	4	Present key elements of study design early in the paper	5
Setting	5	Describe the setting, locations, and relevant dates, including periods of recruitment, exposure, follow-up, and data collection	5
Participants	6	(*a*) Give the eligibility criteria, and the sources and methods of selection of participants. Describe methods of follow-up	5-7
		(*b*) For matched studies, give matching criteria and number of exposed and unexposed	N/A
Variables	7	Clearly define all outcomes, exposures, predictors, potential confounders, and effect modifiers. Give diagnostic criteria, if applicable	5-6
Data sources/ measurement	8*	For each variable of interest, give sources of data and details of methods of assessment (measurement). Describe comparability of assessment methods if there is more than one group	*5-8*
Bias	9	Describe any efforts to address potential sources of bias	5-8
Study size	10	Explain how the study size was arrived at	5-6
Quantitative variables	11	Explain how quantitative variables were handled in the analyses. If applicable, describe which groupings were chosen and why	6-7
Statistical methods	12	(*a*) Describe all statistical methods, including those used to control for confounding	7-9
		(*b*) Describe any methods used to examine subgroups and interactions	7-9
		(*c*) Explain how missing data were addressed	7-9
		(*d*) If applicable, explain how loss to follow-up was addressed	7-9
		(*e*) Describe any sensitivity analyses	7-9 (Appendix Figures 4-8)
**Results**			

Participants	13*	(a) Report numbers of individuals at each stage of study—eg numbers potentially eligible, examined for eligibility, confirmed eligible, included in the study, completing follow-up, and analysed	8-9
		(b) Give reasons for non-participation at each stage	8-9
		(c) Consider use of a flow diagram	N/A
Descriptive data	14*	(a) Give characteristics of study participants (eg demographic, clinical, social) and information on exposures and potential confounders	8-10
		(b) Indicate number of participants with missing data for each variable of interest	8-10
		(c) Summarise follow-up time (eg, average and total amount)	8-11
Outcome data	15*	Report numbers of outcome events or summary measures over time	8-11
Main results	16	(*a*) Give unadjusted estimates and, if applicable, confounder-adjusted estimates and their precision (eg, 95% confidence interval). Make clear which confounders were adjusted for and why they were included	10-11
		(*b*) Report category boundaries when continuous variables were categorized	10-11
		(*c*) If relevant, consider translating estimates of relative risk into absolute risk for a meaningful time period	10-11
Other analyses	17	Report other analyses done—eg analyses of subgroups and interactions, and sensitivity analyses	10-11
**Discussion**			
Key results	18	Summarise key results with reference to study objectives	12-13
**Limitations**			
Interpretation	20	Give a cautious overall interpretation of results considering objectives, limitations, multiplicity of analyses, results from similar studies, and other relevant evidence	13
Generalisability	21	Discuss the generalisability (external validity) of the study results	13
**Other information**			
Funding	22	Give the source of funding and the role of the funders for the present study and, if applicable, for the original study on which the present article is based	8,14

*Give information separately for cases and controls in case-control studies and, if applicable, for exposed and unexposed groups in cohort and cross-sectional studies.

Supplementary figure 1. Risk of recurrent vascular events by degree of asymptomatic ipsilateral carotid stenosis in 207 patients with asymptomatic carotid stenosis in the Oxford Vascular Study.

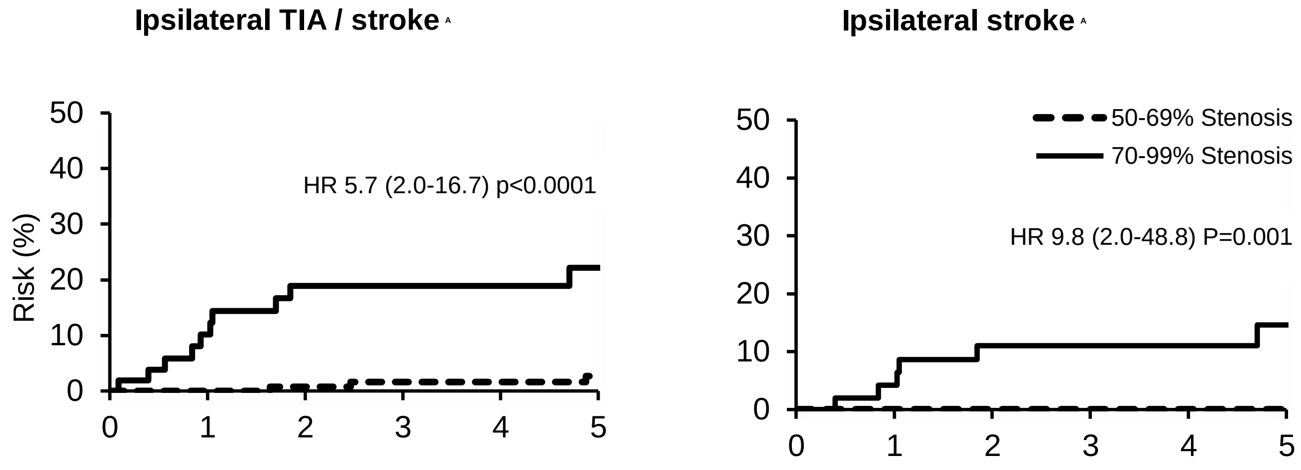

50-69	154	140	128	117	96	88	154	139	125	113	93	87
70-99	53	43	35	32	29	25	53	43	34	30	28	24

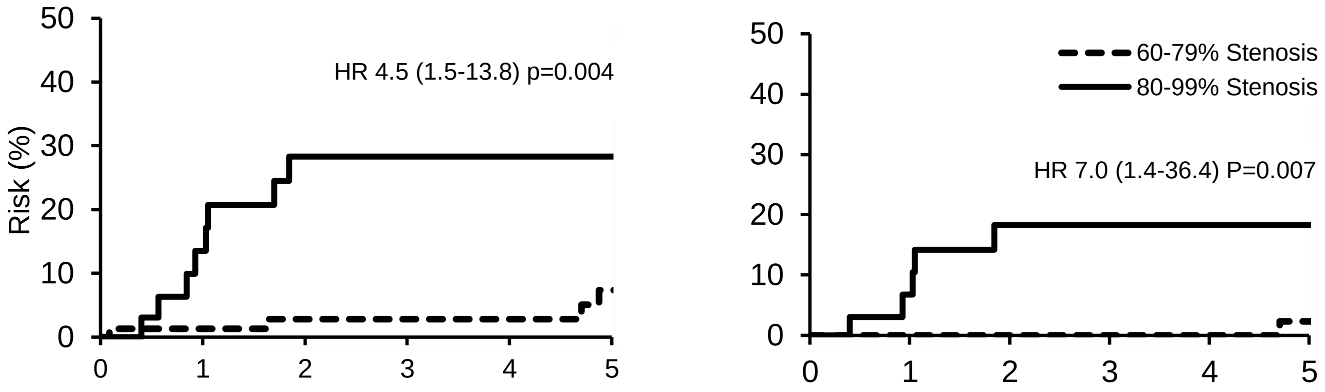

60-79%	76	70	62	58	49	43	76	70	62	58	49	43
80-99%	34	25	19	16	14	14	34	26	20	17	14	14

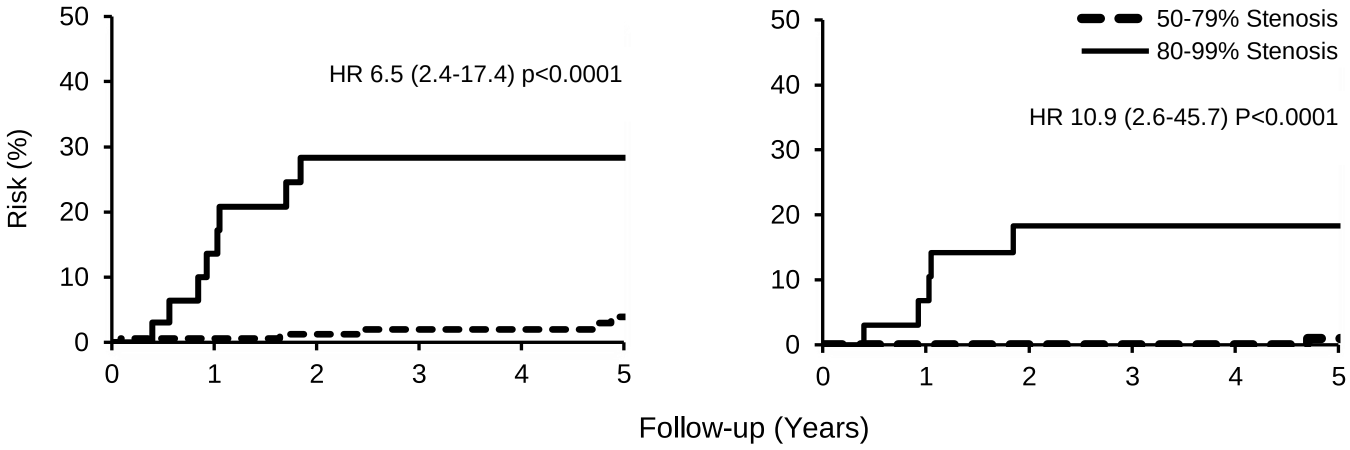

50-79%	173	158	144	133	111	98	173	157	144	134	112	100
80-99%	34	25	19	16	14	14	34	26	20	17	14	14

The exact range and cut-points used to report the stenosis/risk data in trials and previous studies varies with some defining severe stenosis as 70-99% and some as 80-99%. We therefore analysed various cut-points in The Oxford Vascular Study.

Supplementary Figure 2. Risk of events by degree of asymptomatic ipsilateral carotid stenosis excluding patients with baseline AF (n=51 excluded)

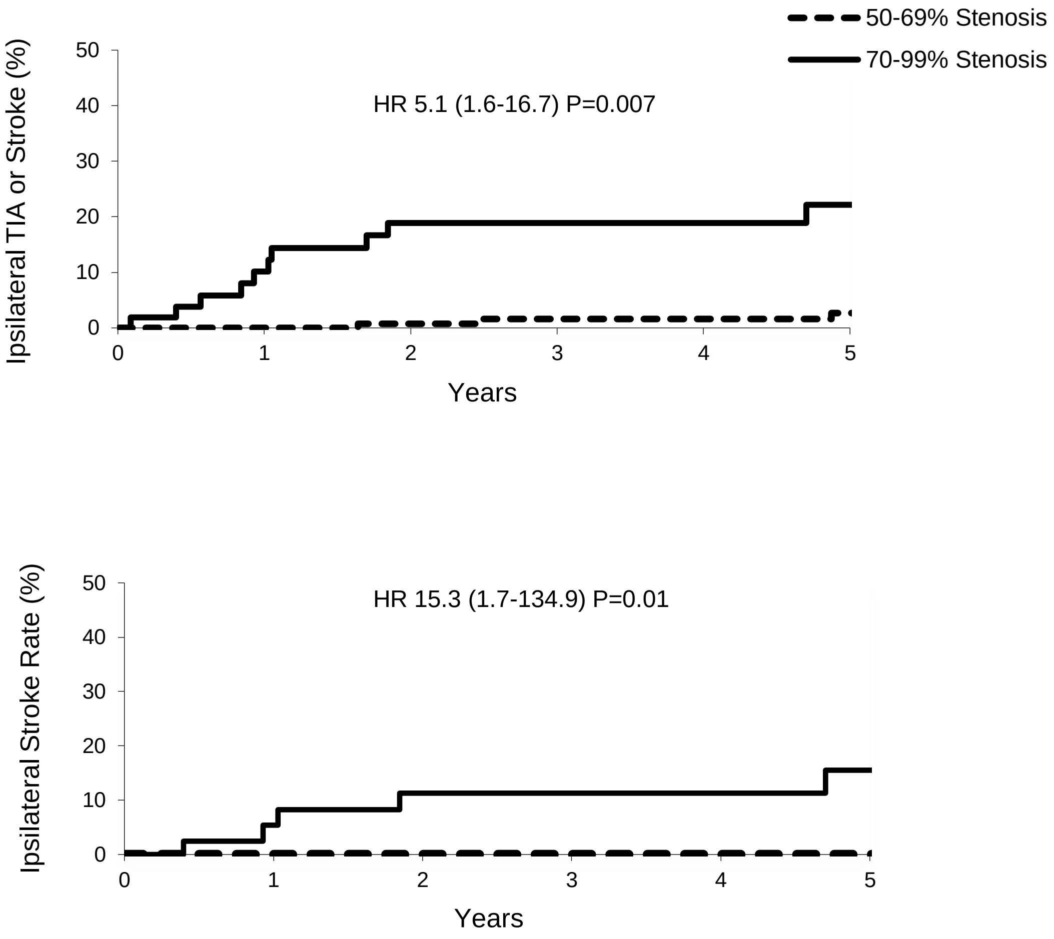

Numbers At Risk

50-69%	114	106	98	91	79	72
70-99%	42	34	29	27	23	21

Supplementary Figure 3. PRISMA diagram for Systematic Review and Meta-analysis

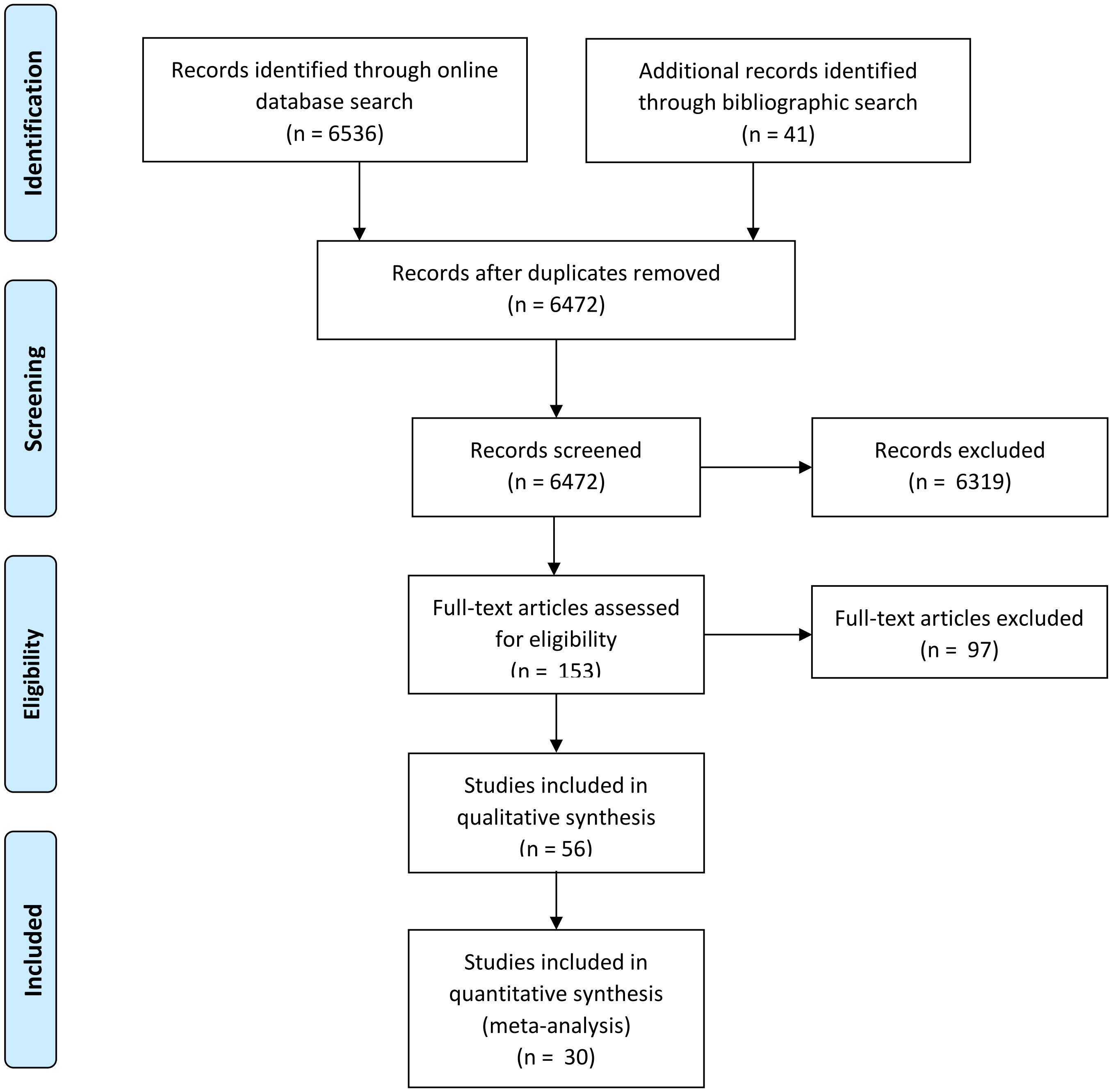

Supplementary Appendix 4

Systematic Review Reference List

1. Halliday A, Mansfield A, Marro J, et al. Prevention of disabling and fatal strokes by successful carotid endarterectomy in patients without recent neurological symptoms: randomised controlled trial. Lancet 2004;363:1491-502.
2. Endarterectomy for asymptomatic carotid artery stenosis. Executive Committee for the Asymptomatic Carotid Atherosclerosis Study. JAMA 1995;273:1421-8.
3. Halliday A, Harrison M, Hayter E, Kong X, Mansfield A, Marro J, et al. 10-year stroke prevention after successful carotid endarterectomy for asymptomatic stenosis (ACST-1): a multicentre randomised trial. Lancet 2010;376:1074-84
4. Norris JW, Zhu CZ, Bornstein NM, Chambers BR. Vascular risks of asymptomatic carotid stenosis. Stroke 1991;22:1485-90.
5. Mackey AE, Abrahamowicz M, Langlois Y, et al. Outcome of asymptomatic patients with carotid disease. Asymptomatic Cervical Bruit Study Group. Neurology 1997;48:896-903.
6. Longstreth WT, Jr., Shemanski L, Lefkowitz D, O'Leary DH, Polak JF, Wolfson SK, Jr. Asymptomatic internal carotid artery stenosis defined by ultrasound and the risk of subsequent stroke in the elderly. The Cardiovascular Health Study. Stroke 1998;29:2371-6.
7. Abbott AL, Chambers BR, Stork JL, Levi CR, Bladin CF, Donnan GA. Embolic signals and prediction of ipsilateral stroke or transient ischemic attack in asymptomatic carotid stenosis: a multicenter prospective cohort study. Stroke 2005;36:1128-33.
8. Goessens BM, Visseren FL, Kappelle LJ, Algra A, van der Graaf Y. Asymptomatic carotid artery stenosis and the risk of new vascular events in patients with manifest arterial disease: the SMART study. Stroke 2007;38:1470-5.
9. Dick P, Sherif C, Sabeti S, Amighi J, Minar E, Schillinger M. Gender differences in outcome of conservatively treated patients with asymptomatic high grade carotid stenosis. Stroke 2005;36:1178-83.
10. Nicolaides AN, Kakkos SK, Kyriacou E, et al. Asymptomatic internal carotid artery stenosis and cerebrovascular risk stratification. J Vasc Surg 2010;52:1486-96 e1-5.
11. Markus HS, King A, Shipley M, et al. Asymptomatic embolisation for prediction of stroke in the Asymptomatic Carotid Emboli Study (ACES): a prospective observational study. Lancet Neurol 2010;9:663-71.
12. Madani A, Beletsky V, Tamayo A, Munoz C, Spence JD. High-risk asymptomatic carotid stenosis: ulceration on 3D ultrasound vs TCD microemboli. Neurology 2011;77:744-50.
13. Silvestrini M, Cagnetti C, Pasqualetti P, et al. Carotid wall thickness and stroke risk in patients with asymptomatic internal carotid stenosis. Atherosclerosis 2010;210:452-7.
14. Durham CA, Ehlert BA, Agle SC, et al. Role of statin therapy and angiotensin blockade in patients with asymptomatic moderate carotid artery stenosis. Ann Vasc Surg 2012;26:344-52.
15. Silvestrini M, Altamura C, Cerqua R, Pasqualetti P, Viticchi G, Provinciali L, Paulon L and Vernieri F. Ultrasonographic markers of vascular risk in patients with asymptomatic carotid stenosis. Journal of cerebral blood flow and metabolism : official journal of the International Society of Cerebral Blood Flow and Metabolism. 2013;33:619-24.

16. Sabeti S, Schlager O, Exner M, et al. Progression of carotid stenosis detected by duplex ultrasonography predicts adverse outcomes in cardiovascular high-risk patients. Stroke. Nov 2007;38(11):2887-2894.
17. Conrad MF, Boulom V, Mukhopadhyay S, Garg A, Patel VI and Cambria RP. Progression of asymptomatic carotid stenosis despite optimal medical therapy. Journal of vascular surgery. 2013;58:128-35 e1
18. Hicks CW, Talbott K, Canner JK, Qazi U, Arhuidese I, Glebova NO, Freischlag JA, Perler BA and Malas MB. Risk of disease progression in patients with moderate asymptomatic carotid artery stenosis: implications of tobacco use and dual antiplatelet therapy. Annals of vascular surgery. 2015;29:1-8
19. Wang D, Wang J, Jin C, Ji R, Wang A, Li X, Gao X, Wu S, Zhou Y and Zhao X. Asymptomatic Extracranial Artery Stenosis and the Risk of Cardiovascular and Cerebrovascular Diseases. Scientific reports. 2016;6:33960
20. Hobson RW, 2nd, Weiss DG, Fields WS, et al. Efficacy of carotid endarterectomy for asymptomatic carotid stenosis. The Veterans Affairs Cooperative Study Group. N Engl J Med 1993;328:221-7.
21. Inzitari D, Eliasziw M, Gates P, et al. The causes and risk of stroke in patients with asymptomatic internal-carotid-artery stenosis. North American Symptomatic Carotid Endarterectomy Trial Collaborators. N Engl J Med 2000;342:1693-700.
22. AbuRahma, A. F., Cook, C. C., Metz, M. J., Wulu, J. T. & Bartolucci, A. Natural history of carotid artery stenosis contralateral to endarterectomy: results from two randomized prospective trials. *J. Vasc. Surg.* **38,** 1154–1160 (2003)
23. Autret, A., Saudeau, D., Bertrand, P. & Pourcelot, L. Stroke risk in patients with carotid stenosis. *Lancet* (1987).
24. Ballotta, E. *et al.* Progression of atherosclerosis in asymptomatic carotid arteries after contralateral endarterectomy: A 10-year prospective study. *J. Vasc. Surg.* **45,** 516–522 (2007).
25. Ballotta, E. *et al.* Carotid endarterectomy for asymptomatic carotid stenosis in the very elderly. *J. Vasc. Surg.* **61,** 382–388 (2015).
26. Bock, R. W. *et al.* The natural history of asymptomatic carotid artery disease. *J. Vasc. Surg.* **17,** 160-9–1 (1993).
27. Bogousslavsky, J., Despland, P. A. & Regli, F. Asymptomatic tight stenosis of the internal carotid artery: long-term prognosis. *Neurology* **36,** 861–3 (1986).
28. Buratti, L. *et al.* Sex-associated differences in the modulation of vascular risk in patients with asymptomatic carotid stenosis. *J. Cereb. Blood Flow Metab.* **35,** 684–8 (2015).
29. den Hartog, A. G. *et al.* Asymptomatic Carotid Artery Stenosis and the Risk of Ischemic Stroke According to Subtype in Patients With Clinical Manifest Arterial Disease. *Stroke* **44,** 1002–1007 (2013).
30. Divya, K. P., Sandeep, N., Sarma, S. & Sylaja, P. N. Risk of Stroke and Cardiac Events in Medically Treated Asymptomatic Carotid Stenosis. *J. Stroke Cerebrovasc. Dis.* **24,** 2149–2153 (2015).
31. Dua, A. *et al.* Asymptomatic 50% to 75% internal carotid artery stenosis in 288 patients: risk factors for disease progression and ipsilateral neurological symptoms. *Perspect. Vasc. Surg. Endovasc. Ther.* **24,** 165–70 (2012).
32. Durward, Q., Ferguson, G. & Barr, H. The natural history of asymptomatic carotid bifurcation plaques. *Stroke* (1982).
33. Ford, C. S., Frye, J. L., Toole, J. F. & Lefkowitz, D. Asymptomatic carotid bruit and stenosis. A prospective follow-up study. *Arch. Neurol.* **43,** 219–22 (1986).
34. Furui, E. *et al.* Abstract 162: Ipsilateral Ischemic Stroke in Japanese Patients With Asymptomatic Carotid Stenosis on Recent Medical Treatment. *Stroke* **45,** (2014).
35. Grønholdt, M. L., Nordestgaard, B. G., Schroeder, T. V, Vorstrup, S. & Sillesen, H. Ultrasonic echolucent carotid plaques predict future strokes. *Circulation* **104,** 68–73 (2001).
36. Gur, A. Y., Bova, I. & Bornstein, N. M. Is Impaired Cerebral Vasomotor Reactivity a Predictive Factor of Stroke in Asymptomatic Patients? *Stroke* **27,** (1996).
37. Hatsukami, T. S., Healy, D. A., Primozich, J. F., Bergelin, R. O. & Strandness, D. E. Fate of the carotid artery contralateral to endarterectomy. *J. Vasc. Surg.* **11,** 244-50–1 (1990).

38. Johnson, J. M., Kennelly, M. M., Decesare, D., Morgan, S. & Sparrow, A. Natural history of asymptomatic carotid plaque. *Arch. Surg.* **120,** 1010–2 (1985).
39. Kirkpatrick, A. C., Tafur, A. J., Vincent, A. S., Dale, G. L. & Prodan, C. I. Coated-Platelets Improve Prediction of Stroke and Transient Ischemic Attack in Asymptomatic Internal Carotid Artery Stenosis. *Stroke* **45,** 2995–3001 (2014).
40. Mansour, M. A. *et al.* Outcome of moderate carotid artery stenosis in patients who are asymptomatic. *J. Vasc. Surg.* **29,** 217-25–7 (1999).
41. Mansour, M. A. *et al.* The natural history of moderate (50% to 79%) internal carotid artery stenosis in symptomatic, nonhemispheric, and asymptomatic patients. *J. Vasc. Surg.* **21,** 346-56–7 (1995).
42. Markus, H. & Cullinane, M. Severely impaired cerebrovascular reactivity predicts stroke and TIA risk in patients with carotid artery stenosis and occlusion. *Brain* **124,** 457–67 (2001).
43. Mono, M.-L. *et al.* Plaque characteristics of asymptomatic carotid stenosis and risk of stroke. *Cerebrovasc. Dis.* **34,** 343–50 (2012).
44. Nicolaides, A. N. *et al.* Severity of asymptomatic carotid stenosis and risk of ipsilateral hemispheric ischaemic events: results from the ACSRS study. *Eur. J. Vasc. Endovasc. Surg.* **30,** 275–84 (2005).
45. Olin, J. W. *et al.* The natural history of asymptomatic moderate internal carotid artery stenosis by duplex ultrasound. *Vasc. Med.* **3,** 101–108 (1998).
46. Park, Y.-J., Kim, D.-I., Kim, G.-M., Kim, D.-K. & Kim, Y. Natural History of Asymptomatic Moderate Carotid Artery Stenosis in the Era of Medical Therapy. *World Neurosurg.* **91,** 247–253 (2016).
47. Rockman, C. B. *et al.* Natural history and management of the asymptomatic, moderately stenotic internal carotid artery. *J. Vasc. Surg.* **25,** 423–431 (1997).
48. Roederer, G. O. *et al.* The natural history of carotid arterial disease in asymptomatic patients with cervical bruits. *Stroke* **15,** 605–13 (1984).
49. Satiani, B., Porter, R. M., Biggers, K. M. & Das, B. M. Natural History of Nonoperated, Significant Carotid Stenosis. *Ann. Vasc. Surg.* **2,** 271–278 (1988).
50. Sato, K. *et al.* Low Stroke Rate of Carotid Stenosis Under the Guideline-Oriented Medical Treatment Compared With Surgical Treatment. *Int. Heart J.* **57,** 80–86 (2016).
51. Shanik, G. D., Moore, D. J., Leahy, A., Grouden, M. C. & Colgan, M.-P. Asymptomatic carotid stenosis: A benign lesion? *Eur. J. Vasc. Surg.* **6,** 10–15 (1992).
52. Siebler, M. *et al.* Cerebral microembolism and the risk of ischemia in asymptomatic high-grade internal carotid artery stenosis. *Stroke* **26,** 2184–6 (1995).
53. Silvestrini, M. *et al.* Impaired cerebral vasoreactivity and risk of stroke in patients with asymptomatic carotid artery stenosis. *JAMA* **283,** 2122–7 (2000).
54. Singh, N. *et al.* Moderate carotid artery stenosis: MR imaging-depicted intraplaque hemorrhage predicts risk of cerebrovascular ischemic events in asymptomatic men. *Radiology* **252,** 502–8 (2009).
55. Spence, J. D. *et al.* Effects of Intensive Medical Therapy on Microemboli and Cardiovascular Risk in Asymptomatic Carotid Stenosis. *Arch. Neurol.* **67,** 180 (2010).
56. Takaya, N. *et al.* Association Between Carotid Plaque Characteristics and Subsequent Ischemic Cerebrovascular Events: A Prospective Assessment With MRI--Initial Results. *Stroke* **37,** 818–823 (2006).
57. Tong, Y. & Royle, J. Outcome of patients with symptomless carotid bruits: a prospective study. *Cardiovasc. Surg.* **4,** 174–80 (1996).
58. Zhang, C. *et al.* Microembolic signals and carotid plaque characteristics in patients with asymptomatic carotid stenosis. *Scand. Cardiovasc. J.* **43,** 345–51 (2009).

Supplementary Table 1: Characteristics of studies describing prognosis in non-operated asymptomatic carotid stenosis

STUDY	START RECRUIT	FINISH RECRUIT	MEDIAN RECRUIT	PUBLICATION	MEAN AGE	NUMBER OF PATIENTS	IPSILATERAL STROKE RATE	STENOSIS	STATIN (%)	TYPE OF COHORT	REGION	REASON FOR RECRUITMENT
**DURWARD**	1971	1981	1976	1982	62	67	0.8	50-99%	0	retrospective	Canada	contralateral CEA
**FORD**	1976	1983	1980	1986	60	36	0.7	50-99%	0	prospective	USA	carotid bruit
**ROEDERER**	1980	1982	1981	1984	63.6	101	1	50-99%	0	prospective	USA	carotid bruit
**BOGOUSSLAVSKY**	1980	1984	1982	1986	60	38	1.7	90-99%	0	prospective	Switzerland	carotid bruit
**AUTRET**	1981	1982	1982	1987	69	113	1.8	50-99%	0	prospective	France	cardiovascular
**JOHNSON**	1981	1984	1983	1985	NG	121	3.3	50-99%	0	prospective	USA	carotid bruit, nonspecific symptoms, routine physical examination
**SATIANI**	1979	1986	1983	1988	NG	103	2.43	50-99%	0	retrospective	USA	carotid bruit
**HATSUKAMI**	NG	NG	1983	1990	65	74	0	50-99%	NG	retrospective	USA	contralateral CEA
**SHANIK**	1981	1984	1983	1992	69	92	2.2	50-99%	0	prospective	Ireland	cardiovascular
**NORRIS**	1982	1986	1984	1991	66.6	177	2.5	50-99%	0	prospective	Canada	asymptomatic cervical bruit
**ECST**	1981	1991	1986	1995	64	127	2.3	50-99%	0	trial	Europe	contralateral TIA or stroke
**HOBSON VA TRIAL**	1983	1991	1987	1993	64.7	233	2.4	50-99%	0	trial	USA	cardiovascular
**BOCK**	1985	1991	1987	1993	68.1	74	3.8	50-99%	0	prospective	Australia	carotid bruit
**TONG**	1980	1993	1987	1996	65	142	0.86	60-99%	0	prospective	Australia	carotid bruit
**ACAS**	1988	1993	1990	1995	66	834	2.3	60-99%	0	trial	USA	carotid bruits, evaluation for peripheral vascular surgery, contralateral CEA
**MANSOUR**	1987	1993	1990	1995	68.6	142	1.3	50-79%	0	retrospective	USA	Carotid buit, preoperative work-up for cardio thoracic surgery, contralateral CEA
**SEIBLER**	NG	NG	1991	1995	65.5	64	3.4	70-90%	0	prospective	Germany	cardiovascular
**MACKEY (ACBS)**	1988	1994	1991	1997	65	330	1.3	50-99%	0	prospective	Canada	carotid bruit
**ROCKMAN**	1990	1992	1991	1997	74.5	425	1.2	50-79%	0	retrospective	USA	bruit, contralateral TIA/stroke,
**LONGSTRETH (CHS)**	1989	1992	1991	1998	73	185	1	70-99%	5.6	prospective	USA	general population screening (aged >65)
**OLIN**	1989	1994	1992	1998	69	465	0.7	60-79%	NG	retrospective	USA	bruit, contralateral CEA, contralateral TIA/stroke, cardiothoracic peroperative workup
**INZITARI (NASCET)**	1988	1997	1992	2000	66	216	3.2	50-99%	0	trial	USA	contralateral TIA or stroke
**MANSOUR**	1992	1996	1994	1999	70.6	344	1.9	50-79%	NG	prospective	USA	carotid bruits, cardiothoracic peroperative workup
**GUR**	1995	1995	1995	1996	69	44	2.2	70-99%	0	prospective	Isreal	NG

**GRONHOLDT**	1994	1996	1995	2001	64	111	3.5	50-99%	0	prospective	Denmark	carotid bruit, nonhemispheric symptoms such as dizziness and vertigo, contralateral TIA/stroke
**ABURAHMA**	1991	2000	1995	2003	68	101	1.5	50-99%	NG	prospective	USA	contralateral CEA
**SILVERSTRINI**	1996	1998	1997	2000	71.1	94	5.4	50-99%	32	prospective	Italy	cardiovascular
**MARKUS**	NG	NG	1997	2001	NG	59	1	70-99%	NG	prospective	UK	cardiovascular
**HALLIDAY (ACST)**	1993	2003	1998	2004	68	1560	1.2	60-99%	38	trial	Europe	cardiovascular
**ABBOTT (ASED)**	1996	2000	1998	2005	74	202	1.2	60-99%	75	prospective	Australia	carotid bruit, extracerebral vascular disease, or cerebrovascular symptoms
**DICK**	1997	2002	2000	2005	73	525	2.1	70-99%	62	prospective	Austria	cardiovascular
**NICOLAIDES (ACSRS)**	1998	2002	2000	2005	70	805	1.4	50-99%	25	prospective	UK	cardiovascular
**BALLOTTA**	1995	2004	2000	2007	71	98	0.7	50-99%	NG	prospective	Italy	contralateral CEA
**DURHAM**	1998	2001	2000	2012	69	366	1.5	50-79%	63	retrospective	USA	carotid bruit, vertebrobasilar symptoms, hypertension, preoperative workup, screening programs
**TAKAYA**	2000	2004	2002	2006	71.1	154	1.2	50-79%	64	prospective	USA	cardiovascular
**SABETI**	2002	2003	2002	2007	71.8	376	2.3	50-99%	59	prospective	Austria	cardiovascular
**MARKUS (ACES)**	1999	2007	2003	2010	71.5	467	0.7	70-99%	65	prospective	Internationa l	cardiovascular
**SPENCE**	2000	2007	2004	2010	70	468	0.6	60-99%	44.1	prospective	Canada	carotid bruit
**DEN HARTOG (SMART)**	1996	2012	2004	2013	65.2	293	0.3	50-99%	60	prospective	Netherlands	cardiovascular
**SINGH**	2003	2006	2005	2009	92	75	1.3	50-70%	72	retrospective	Canada	cardiovascular
**SILVESTRINI**	2004	2006	2005	2010	75	162	2.8	50-99%	49	prospective	Italy	cardiovascular
**CONRAD**	2005	2006	2005	2013	72.5	794	1.3	50-69%	87.1	retrospective	USA	cardiovascular
**ZHANG**	NG	NG	2006	2009	65.5	62	1.6	50-99%	NG	prospective	China	cardiovascular
**BURATTI**	2003	2009	2006	2015	72.5	1109	1.8	50-99%	44	prospective	Italy	cardiovascular
**PARK**	2003	2008	2006	2016	67.7	124	0.6	50-69%	89	retrospective	South Korea	cardiovascular
**DUA**	2008	2008	2008	2012	75	288	1	50-75%	63	retrospective	USA	cardiovascular
**MONO**	2007	2009	2008	2012	68.7	60	2.6	50-99%	82	prospective	Switzerland	cardiovascular
**HICKS**	2005	2012	2008	2015	70.5	258	0.9	50-99%	75	retrospective	USA	cardiovascular
**DIVYA**	2005	2013	2009	2015	63.4	75	0	50-99%	97	prospective	India	contralateral TIA or stroke
**BALLOTTA**	2005	2012	2009	2015	84.9	54	1.3	60-99%	81.5	retrospective	Italy	patients denied CEA
**SATO**	2006	2013	2010	2016	77.5	67	0	50-99%	70	retrospective	Japan	cardiovascular
**OXVASC**	2002	2017	2010	2021	77.5	207	0.7	50-99%	89	prospective	UK	contralateral TIA or stroke
**FURUI**	2009	2013	2011	2014	76	105	0.7	50-99%	NG	prospective	Japan	NG
**KIRKPATRICK**	2010	2013	2012	2014	66.1	118	2.7	50-99%	74	prospective	USA	cardiovascular

N G: not given

Supplementary figure 3. Ratio of ipsilateral stroke risk in medically treated patients with asymptomatic carotid stenosis by degree of stenosis in all studies published since 1980 stratified by the nature of the study design - observational cohort studies versus the medical treatment group of randomised trials

Study	≥70% stenosis Strokes / Participants	<70% stenosis Strokes/ Participants	OR	95% CI
**COHORTS: No Previous cerebrovascular Events**				
Johnson	12/121	3/176	6.3	1.8-23.0
Hertzer	18 / 69	3 / 88	10.0	2.8-35.6
Chambers	11 / 113	5 / 157	3.3	1.1-9.7
O'Holleran	15 / 120	4 / 173	6.0	2.0-18.7
Autret	2 / 39	2 / 75	2.0	0.3-14.6
Norris	15 / 177	8 / 216	2.4	1.0-5.8
Shanik	4 / 58	2 / 34	1.2	0.2-6.8
SMART	4 / 193	1 / 100	2.1	0.2-19.0
Silvestrini	25 / 207	14 / 414	3.9	2.0-7.7
POOLED	106 / 1097	42 / 1433	3.9	2.7-5.7
**COHORTS: Previous cerebrovascular Events**				
ECST	9 / 56	2 / 71	6.6	1.4-32.0
NASCET	19 / 103	29 / 221	1.5	0.8-2.8
ACSRS	24 / 453	12 / 352	1.6	0.8-3.2
Sabeti	11 / 121	17 / 255	1.4	0.6-3.1
Buratti	28 / 207	34 / 902	4.0	2.4-6.8
OXVASC	6 / 53	2 / 154	9.7	1.9-49.7
POOLED	97 / 993	96 / 1955	2.3	1.7-3.2
**ALL COHORTS**	203 / 2090	138 / 3388	2.9	2.3-3.7
**TRIALS**				
VA	10 / 108	12 / 125	1.0	0.4-2.3
ACAS	8/182	8/131	0.7	0.3-1.9
ACST-1	165/1398	23/162	0.8	0.5-1.3
**ALL TRIALS**	183 / 1688	43 / 418	0.8	0.6-1.2

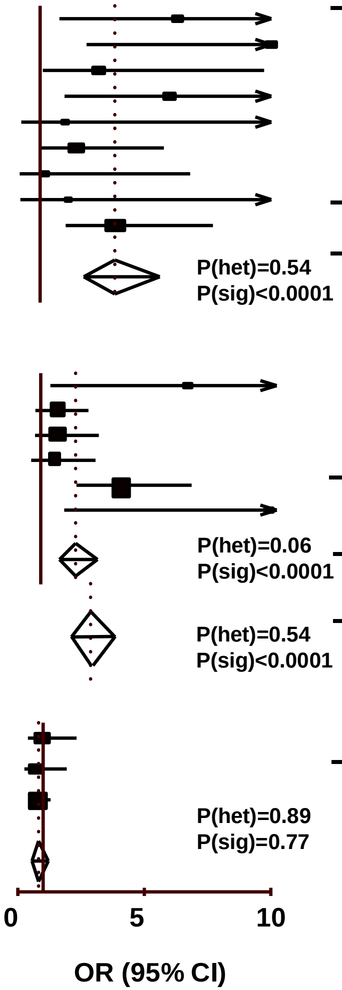

Study	≥80% stenosis Strokes / Participants	<80% stenosis Strokes/ Participants	OR	95% CI
**COHORTS: No Previous cerebrovascular Events**				
Roederer	4 / 28	0 / 72	24.3	1.2-489.7
Hennerici	3 / 36	4 / 199	4.4	0.9-20.7
Bock	1 / 7	3 / 46	2.4	0.2-26.8
Mackey	11 / 113	12 / 244	2.1	0.9-4.9
Silvestrini	6 / 34	33 / 587	3.6	1.4-9.3
POOLED	25 / 218	52 / 1148	3.1	1.8-5.5
**COHORTS: Previous cerebrovascular Events**				
Satiani	2 / 24	3 / 66	1.9	0.3-12.2
ECST	3 / 15	8 / 112	3.3	0.8-13.9
NASCET	14 / 74	31 / 221	1.4	0.7-2.9
ACSRS	20 / 328	16 / 477	1.9	1.0-3.7
Buratti	8 / 34	54 / 1075	5.8	2.5-13.5
OXVASC	5 / 34	3 / 173	9.8	2.2-43.1
POOLED	52 / 509	115 / 2124	2.3	1.6-3.4
**ALL COHORTS**	77 / 727	167 / 3272	2.5	1.8-3.5
**TRIALS**				
ACAS	3 / 88	13 / 225	0.6	0.2-2.1
ACST-1	106 / 917	82 / 643	0.9	0.7-1.2
**ALL TRIALS**	109 / 1005	95 / 868	0.9	0.6-1.2

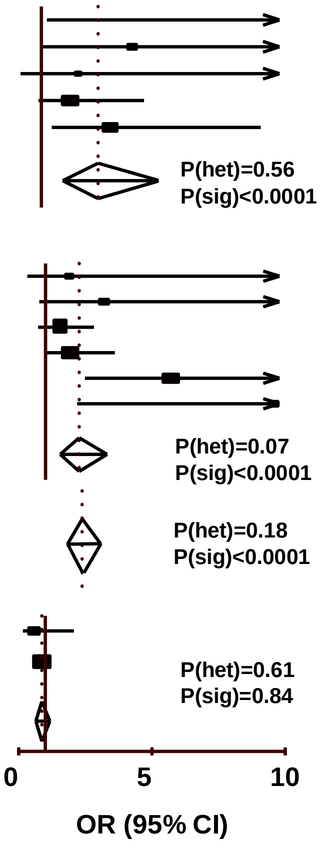

Supplementary Figure 4. Risk of ipsilateral stroke in medically treated patients with asymptomatic carotid stenosis by degree of stenosis in all studies since 1985 stratified by year of study publication – pre-2000 versus post-2000

Study	YR	Severe stenosis Strokes / Participants	Moderate stenosis Strokes/ Participants	OR	95% CI	P_{SIG}	P_{HETERO}
Cohort Studies Pre-2000							
Roederer	1984	4 / 28	0 / 72	24.3	1.2-489.7		
Johnson	1985	12/121	3/176	6.3	1.8-23.0		
Chambers	1986	11 / 113	5 / 157	3.3	1.1-9.7		
Hennerici	1987	3 / 36	4 / 199	4.4	0.9-20.7		
O'Holleran	1987	15 / 120	4 / 173	6.0	2.0-18.7		
Autret	1987	2 / 39	2 / 75	2.0	0.3-14.6		
Satiani	1988	2 / 24	3 / 66	1.9	0.3-12.2		
Norris	1991	15 / 177	8 / 216	2.4	1.0-5.8		
Shanik	1992	4 / 58	2 / 34	1.2	0.2-6.8		
Bock	1993	1 / 7	3 / 46	2.4	0.2-26.8		
ECST	1995	9 / 56	2 / 71	6.6	1.4-32.0		
Mackey	1997	11 / 113	12 / 244	2.1	0.9-4.9		
POOLED		89 / 892	48 / 1529	3.4	2.3-4.9	<0.0001	0.69
Cohort Studies Post-2000							
NASCET	2000	19 / 103	29 / 221	1.5	0.8-2.8		
ACSRS	2005	24 / 453	12 / 352	1.6	0.8-3.2		
Sabeti	2007	11 / 121	17 / 255	1.4	0.6-3.1		
SMART	2013	4 / 193	1 / 100	2.1	0.2-19.0		
Silvestrini	2013	25 / 207	14 / 414	3.9	2.0-7.7		
Buratti	2015	28 / 207	34 / 902	4	2.4-6.8		
OXVASC	2021	6 / 53	2 / 154	9.7	1.9-49.7		
POOLED		117 / 1337	109 / 2398	2.4	1.8-3.2	<0.0001	0.05

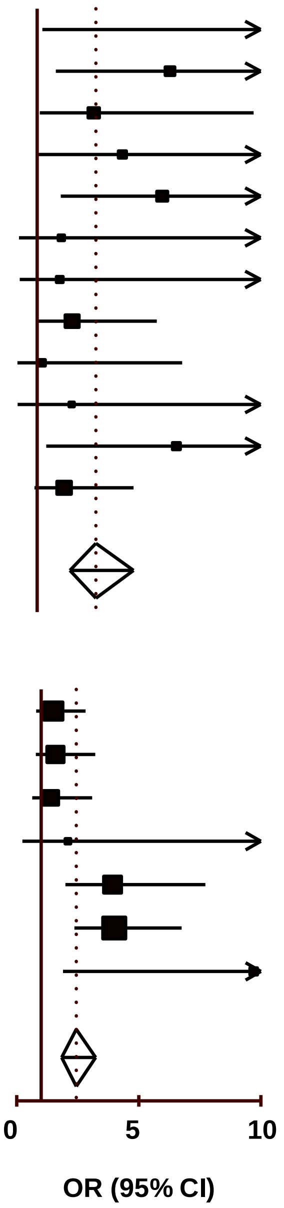

	All cohort studies	Pre-2000 studies	Post-2000 Studies
OR, P_{SIG}	2.7, <0.0001	3.4, <0.0001	2.4, <0.0001
$P_{HETEROGENEITY}$	0.20	0.69	0.05

Supplementary Figure 5. Risk of ipsilateral stroke in medically treated patients with asymptomatic carotid stenosis by degree of stenosis in all studies since 1985 stratified by statin usage– <50% of cohort on treatment versus ≥50% on treatment

Study	Severe stenosis Strokes / Participants	Moderate stenosis Strokes/ Participants	OR	95% CI	P_{SIG}	P_{HETERO}
**<50% Statin Therapy**						
Roederer	4 / 28	0 / 72	24.3	1.2-489.7		
Johnson	12/121	3/176	6.3	1.8-23.0		
Chambers	11 / 113	5 / 157	3.3	1.1-9.7		
Hennerici	3 / 36	4 / 199	4.4	0.9-20.7		
O'Holleran	15 / 120	4 / 173	6.0	2.0-18.7		
Autret	2 / 39	2 / 75	2.0	0.3-14.6		
Satiani	2 / 24	3 / 66	1.9	0.3-12.2		
Norris	15 / 177	8 / 216	2.4	1.0-5.8		
Shanik	4 / 58	2 / 34	1.2	0.2-6.8		
Bock	1 / 7	3 / 46	2.4	0.2-26.8		
ECST	9 / 56	2 / 71	6.6	1.4-32.0		
Mackey	11 / 113	12 / 244	2.1	0.9-4.9		
NASCET	19 / 103	29 / 221	1.5	0.8-2.8		
ACSRS	24 / 453	12 / 352	1.6	0.8-3.2		
Silvestrini	25 / 207	14 / 414	3.9	2.0-7.7		
Buratti	28 / 207	34 / 902	4.0	2.4-6.8		
POOLED	185 / 1862	137 / 3418	2.9	2.3-3.6	<0.0001	0.28
**≥50% Statin Therapy**						
Sabeti	11 / 121	17 / 255	1.4	0.6-3.1		
SMART	4 / 193	1 / 100	2.1	0.2-19.0		
OXVASC	6 / 53	2 / 154	9.7	1.9-49.7		
POOLED	21 / 367	20 / 509	2.1	1.1-4.0	0.02	0.11

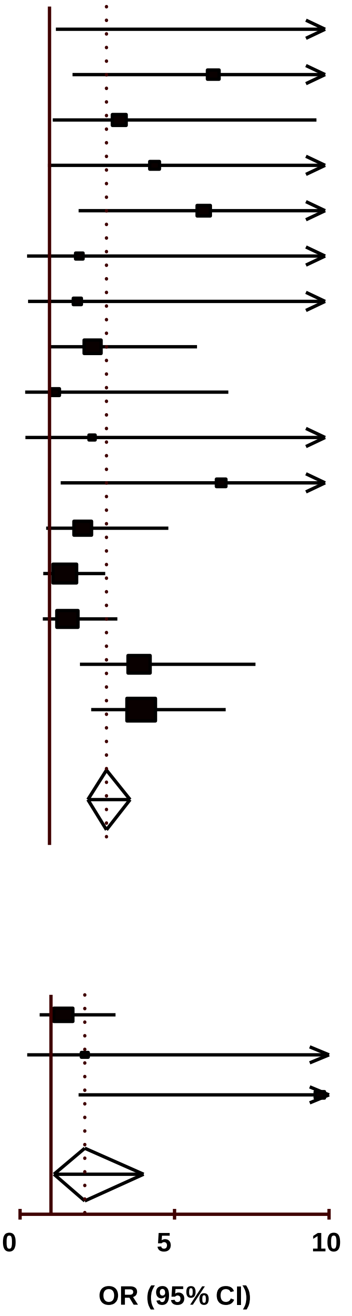

Supplementary Figure 6. Risk of ipsilateral stroke in medically treated patients with asymptomatic carotid stenosis by degree of stenosis in all studies since 1985 stratified by grading of study quality – high versus low-to-medium

Study	YR	Severe stenosis Strokes / Participants	Moderate stenosis Strokes/ Participants	OR	95% CI	P_{SIG}	P_{HETERO}
Cohort Studies High Quality							
Roederer	1984	4 / 28	0 / 72	24.3	1.2-489.7		
Johnson	1985	12/121	3/176	6.3	1.8-23.0		
Chambers	1986	11 / 113	5 / 157	3.3	1.1-9.7		
Hennerici	1987	3 / 36	4 / 199	4.4	0.9-20.7		
O'Holleran	1987	15 / 120	4 / 173	6.0	2.0-18.7		
Autret	1987	2 / 39	2 / 75	2.0	0.3-14.6		
Satiani	1988	2 / 24	3 / 66	1.9	0.3-12.2		
Norris	1991	15 / 177	8 / 216	2.4	1.0-5.8		
Mackey	1997	11 / 113	12 / 244	2.1	0.9-4.9		
Sabeti	2007	11 / 121	17 / 255	1.4	0.6-3.1		
Silvestrini	2013	25 / 207	14 / 414	3.9	2.0-7.7		
Buratti	2015	28 / 207	34 / 902	4	2.4-6.8		
OXVASC	2021	6 / 53	2 / 154	9.7	1.9-49.7		
POOLED		145 / 1359	108 / 3103	3.3	2.6-4.4	<0.0001	0.36
Cohort Studies Low-to-Medium Quality							
Hertzer	1985	18/69	3/88	10.0	2.8-35.6		
Shanik	1992	4 / 58	2 / 34	1.2	0.2-6.8		
Bock	1993	1 / 7	3 / 46	2.4	0.2-26.8		
ECST	1995	9 / 56	2 / 71	6.6	1.4-32.0		
NASCET	2000	19 / 103	29 / 221	1.5	0.8-2.8		
ACSRS	2005	24 / 453	12 / 352	1.6	0.8-3.2		
SMART	2013	4 / 193	1 / 100	2.1	0.2-19.0		
POOLED		79 / 939	52 / 912	2.2	1.5-3.3	<0.0001	0.12

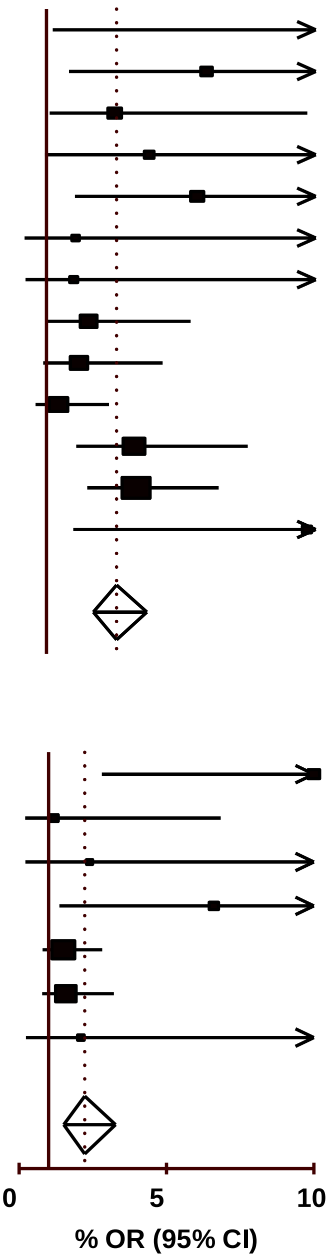

	All cohort studies	High quality	Low-Medium quality
OR, P_{SIG}	2.7, <0.0001	3.3, <0.0001	1.8, 0.0069
$P_{HETEROGENEITY}$	0.20	0.36	0.65

Study quality graded based on STROBE criteria and key criteria required for meta-analysis, including the following items: study objectives stated clearly; patient selection criteria stated clearly; patients enrolled consecutively without predetermined selection; interventions adequately described; outcome definitions provided; rate of dropout or crossover to endarterectomy or stenting of <20%, and outcome ascertainment by a neurologist

Supplementary Figure 7. Annual rate of ipsilateral stroke in medically treated patients with asymptomatic carotid stenosis by degree of stenosis in all studies published since 1980 stratified by the nature of the study design - observational cohort studies versus the medical treatment group of randomised trials

Cohorts (30) refers to all cohort studies reporting risk for 50-69% or 70-99% stenosis;
Cohorts (15) refers to all cohort studies reporting risk in *both* 50-69% and 70-99% stenosis

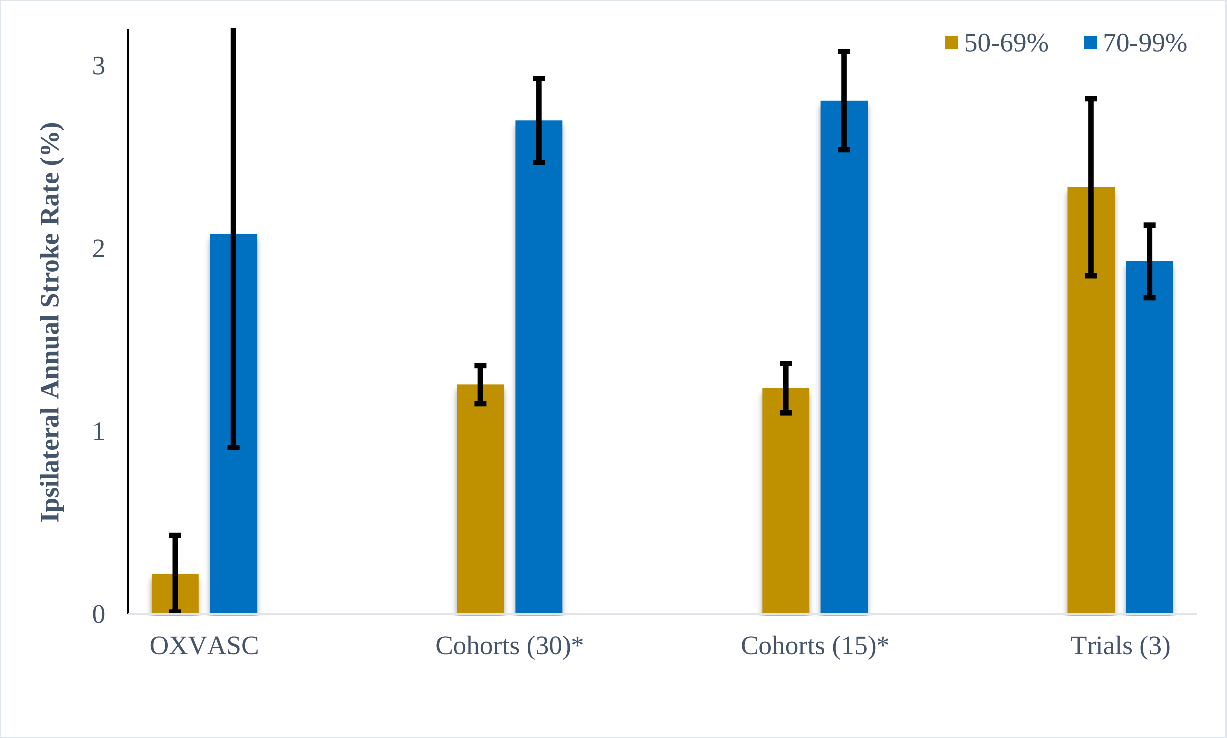

*OXVASC data excluded

Supplementary Figure 8.

A Meta-regression bubble plot of log odd ratio of stroke risk for severe versus moderate carotid stenosis by proportion of cohort with severe stenosis (%) stratified by the nature of the study design - observational cohort studies (blue) versus the medical treatment arm of randomised trials (red) (n=20)

B Time-trend in the proportion of patients with severe asymptomatic stenosis in non-selective cohort studies reporting risk in *both* 50-69% and 70-99% stenosis (n=15) recruiting after 1980

C Trend in stratified ipsilateral stroke risk by proportion of patients with severe stenosis included in non-selective cohort studies (n=15) after 1980

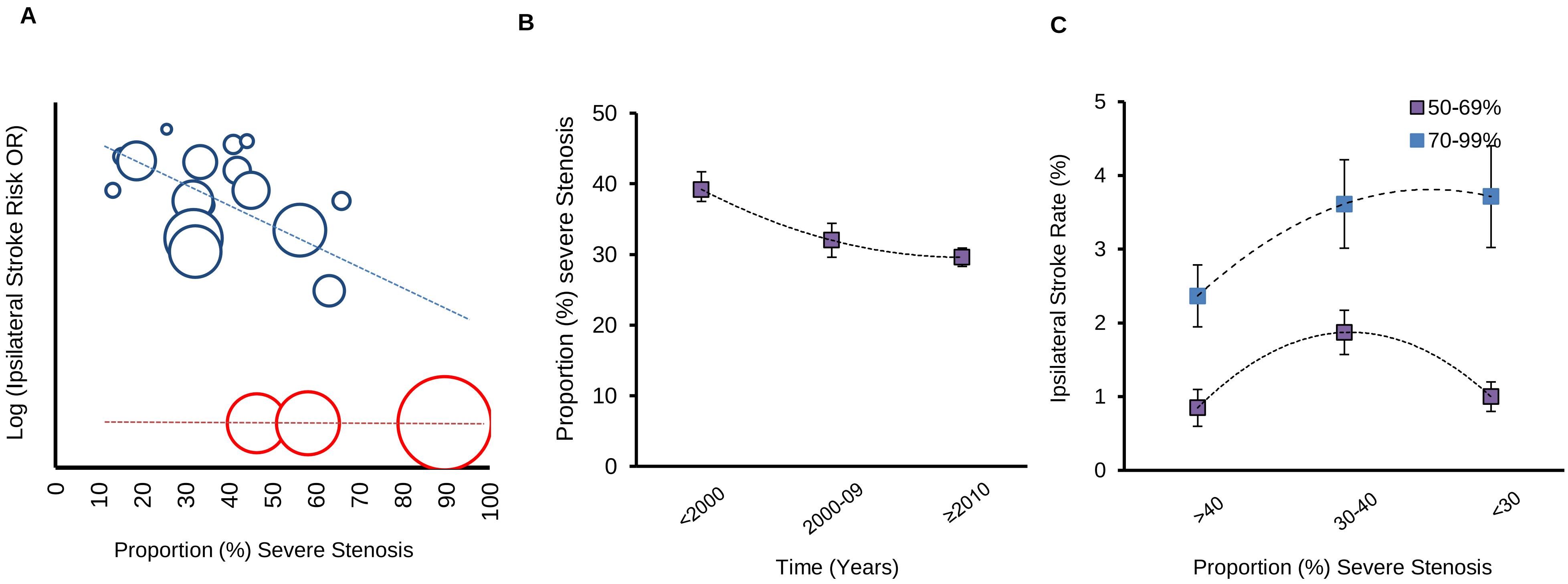

Supplementary Figure 9. Egger Funnel Plot for assessment of potential publication bias, including all cohort studies published since 1980, reporting ipsilateral stroke risk in medically treated patients with asymptomatic carotid stenosis by degree of stenosis

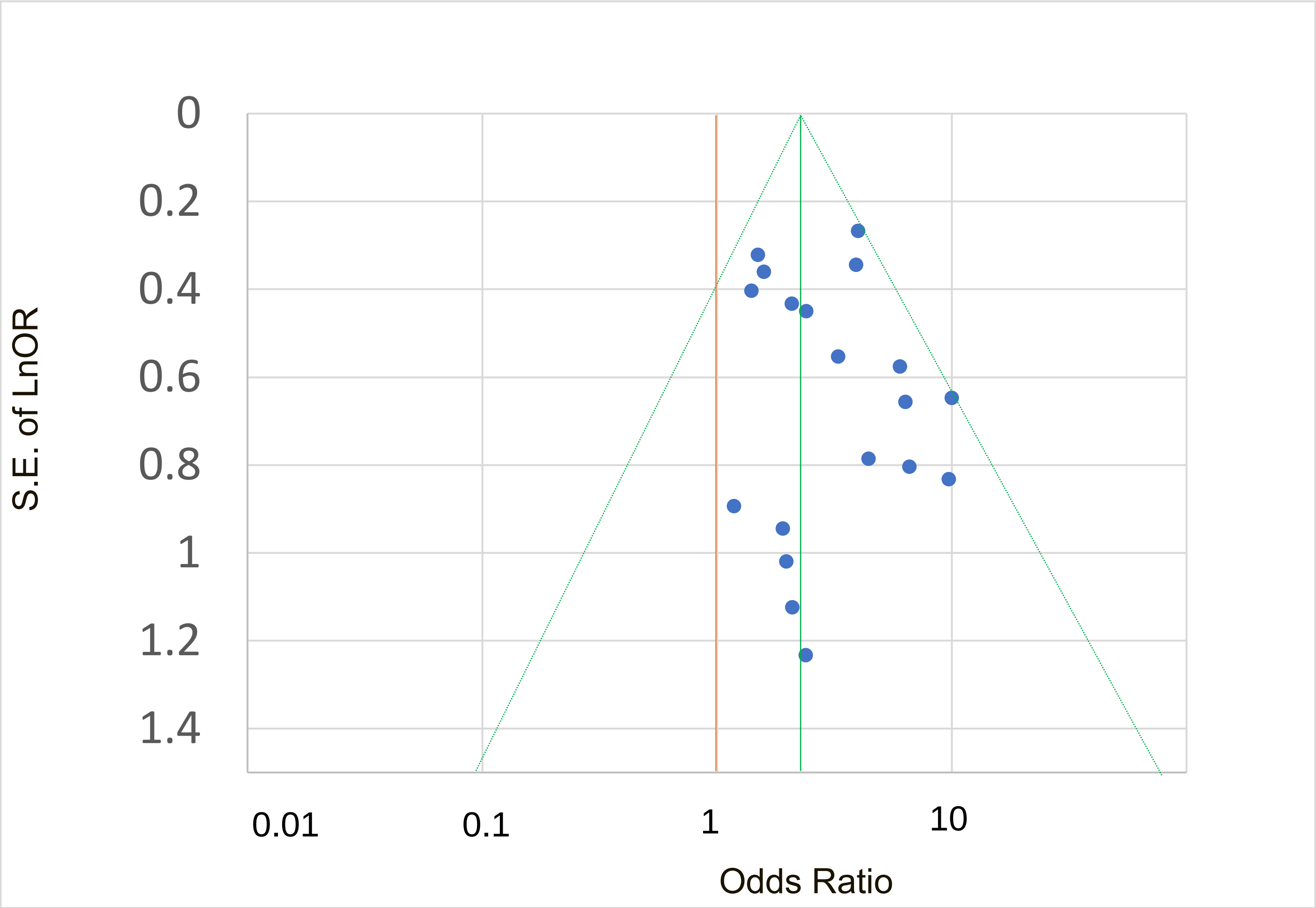

Supplement: Supplementary appendix [file mmc1.pdf]
